# Diagnostic Imaging of Pancreatic and Biliary Involvement in IgG4-Related Disease: Key Imaging Features, Diagnostic Criteria and Differential Diagnosis

**DOI:** 10.3390/diagnostics16121806

**Published:** 2026-06-11

**Authors:** Javier Miguez González, Rafael Oliveira Caiafa, Marc Valls Mellado, Marta López Gómez, Pilar Lozano Arranz, Francesc Calaf Forn, Alona Thomas Martínez, Laura Pelegrí Martínez, Cristina Pallàs Guardiola, Lorena Ivonne Sarati Nieto, Ingrid Carolina Durán Palacios, Angélica María Herrera Pulido, Sergio González Martínez, Jordi Català Forteza

**Affiliations:** 1 Department of Radiology, Complex Hospitalari Universitari Moisès Broggi, Consorci Sanitari Integral, 08970 Sant Joan Despí, Barcelona, Spain; roliveirac@csi.cat (R.O.C.); cpallasg@csi.cat (C.P.G.);; 2 Department of Medicine, Universitat de Barcelona (UB), 08036 Barcelona, Spain; 3 Department of Rheumatology, Complex Hospitalari Universitari Moisès Broggi, Consorci Sanitari Integral, 08970 Sant Joan Despí, Barcelona, Spain; 4 Department of Medicine, Universitat Autònoma de Barcelona (UAB), 08193 Barcelona, Spain; 5 Hepatobiliopancreatic Unit, Department of General and Digestive Surgery, Complex Hospitalari Universitari Moisès Broggi, Consorci Sanitari Integral, 08970 Sant Joan Despí, Barcelona, Spain

**Keywords:** autoimmune pancreatitis, IgG4-related sclerosing cholangitis, IgG4-related disease, diagnostic criteria, differential diagnosis, pancreatic ductal adenocarcinoma, cholangiocarcinoma, magnetic resonance imaging, computed tomography

## Abstract

IgG4-related disease (IgG4-RD) is a systemic fibroinflammatory disorder characterised by elevated serum levels of IgG4 and multiorgan damage. Its diagnosis is challenging and requires a careful integration of clinical, radiological, serological and histological data. Pancreatic and biliary involvement is one of the most common manifestations of IgG4-RD, presenting as type 1 autoimmune pancreatitis (AIP) and IgG4-related sclerosing cholangitis (IgG4-SC), two entities that often occur synchronously and may mimic malignancy in the form of pancreatic ductal adenocarcinoma (PDAC) and cholangiocarcinoma, respectively. The main objective of this article is to illustrate the key imaging features of AIP and IgG4-SC on computed tomography (CT) and magnetic resonance imaging (MRI), providing a comprehensive review of their current diagnostic criteria and discussing their differential diagnosis with other benign and malignant conditions.

## 1. Introduction

**Figure 1:**
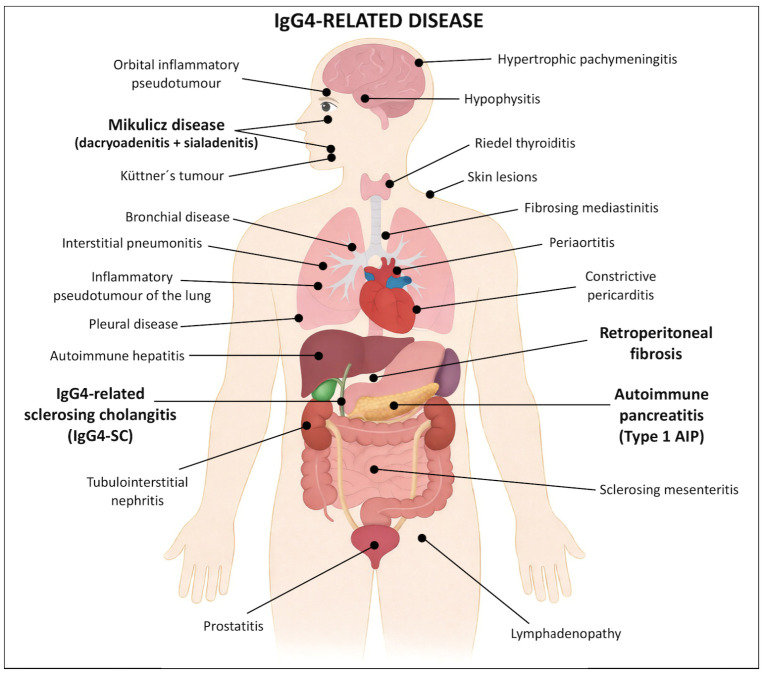
Illustration depicting the broad spectrum of systemic manifestations of IgG4-related disease (IgG4-RD). The most common conditions are highlighted in bold.

IgG4-related disease (IgG4-RD) is a systemic immune-mediated fibroinflammatory disorder, characterised by elevated serum IgG4 levels and encompassing a wide variety of processes involving the central nervous system, head and neck, chest and abdomen (Figure 1). It usually has an insidious clinical onset, resulting in diagnostic delays of several months or even years, which may lead to silent organ damage [1].

Pancreatic and biliary involvement, presenting as type 1 autoimmune pancreatitis (AIP) and IgG4-related sclerosing cholangitis (IgG4-SC), represents a common manifestation within the broad spectrum of IgG4-RD and is considered a hallmark of this disease. The presentation of these two conditions is usually synchronous and can mimic malignancy [2,3]. Therefore, knowledge of their radiological characteristics is essential for an early and accurate diagnosis, allowing for appropriate treatment and avoiding invasive diagnostic procedures and unnecessary surgery.

The main objective of this work is to review the imaging findings and current diagnostic criteria for AIP and IgG4-SC, focusing on the distinctive radiological features that facilitate the differential diagnosis from other benign and malignant conditions.

## 2. Autoimmune Pancreatitis

### 2.1. Clinicopathological Subtypes of AIP

**Figure 2:**
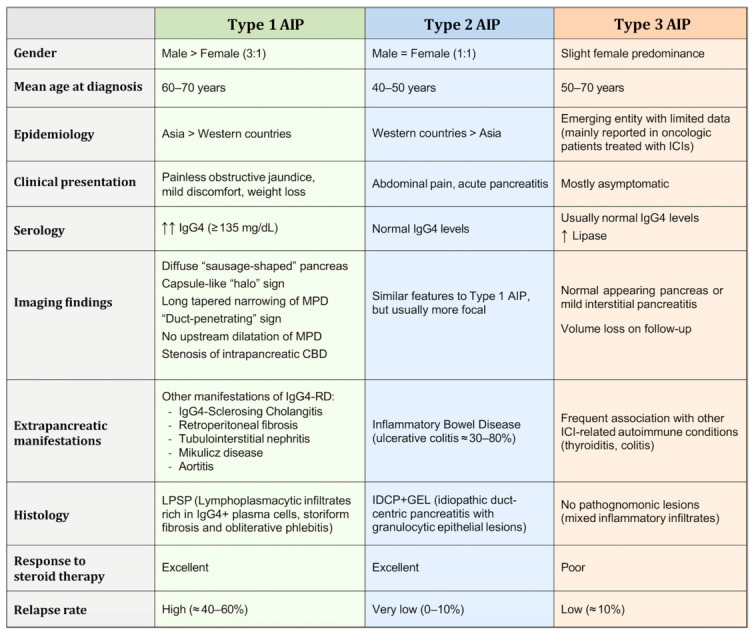
Differential features between the subtypes of autoimmune pancreatitis (AIP). Adapted from Gallo et al. [3], Uchida et al. [7], Vemulapalli et al. [11] and Thomas et al. [18]. MPD = main pancreatic duct; CBD = common bile duct; ICI = immune checkpoint inhibitor.

AIP is a fibroinflammatory pancreatic disease with two main clinicopathological subtypes, type 1 and type 2, which share similar imaging and macroscopic features but differ in their microscopic characteristics. More recently, a third entity has been proposed as an additional subtype of AIP (Figure 2).

#### 2.1.1. Type 1 AIP

This is the most prevalent form of AIP, particularly in Asian populations, where it accounts for the vast majority of reported cases. In a multicentre international analysis, type 1 AIP represented approximately 96% of AIP cases in Asia and 87% in Europe [4]. It is more frequent in males, with a reported male-to-female ratio of 3:1, and usually appears between the sixth and seventh decades of life [1,2,3,4,5,6,7,8,9,10].

The typical clinical presentation consists of painless jaundice with elevated bilirubin, frequently associated with increased serum IgG4 levels (≥135 mg/dL). The tumour marker CA 19-9 may also be mildly increased due to cholestasis, but marked elevations are atypical and should prompt further evaluation for pancreatic malignancy [1,2,3,4,5,6,7,8,9,10,11,12].

Type 1 AIP is associated with extrapancreatic manifestations of IgG4-RD in 60–90% of patients. The most common is synchronous biliary involvement in the form of IgG4-SC, which is observed in up to 50–60% of cases [11,13].

Histologically, the diagnostic hallmark is a pattern called lymphoplasmacytic sclerosing pancreatitis (LPSP). It is characterised by a dense lymphoplasmacytic infiltrate rich in IgG4-positive plasma cells within the inflamed pancreatic tissue, leading to periductal fibrosis with acinar atrophy and obliterative phlebitis [2,3,4,5,6,7,8,9,10].

Glucocorticoids remain the first-line treatment, typically prednisone or an equivalent agent at a dose of 0.5–1.0 mg/kg/day for 2–4 weeks, followed by a gradual taper. A rapid biochemical, clinical and radiological response is usually observed within 1–2 weeks, and both exocrine and endocrine pancreatic dysfunction are often reversible. However, relapses occur in up to 40–60% of patients after withdrawal of steroid therapy. In relapsing disease, re-induction with glucocorticoids is frequently combined with a steroid-sparing agent, such as azathioprine or rituximab. Given this high relapse rate, current guidelines recommend maintenance therapy with low-dose glucocorticoids for 2–3 years [1,2,6,7,8,11].

Given its higher prevalence and close association with IgG4-RD, type 1 AIP is the primary focus of this review, and its imaging features will be discussed in detail in the following sections.

#### 2.1.2. Type 2 AIP

This type of AIP is rare in Asia, accounting for approximately 4% of cases in an international multicentre study, and is more prevalent in Western countries, where it represents up to 13–14% of cases [4]. It shows no sex predominance and typically occurs in younger patients around the fourth decade of life [2,3,4,5,6,7,8].

This entity often presents with a more acute clinical onset characterised by abdominal pain, and acute pancreatitis develops in nearly 50% of patients [3].

Unlike type 1 AIP, it is not associated with IgG4-SC or other extrapancreatic manifestations of IgG4-RD. However, a strong association with inflammatory bowel disease, particularly ulcerative colitis, has been consistently reported, with prevalence rates ranging from 30% to 80% [3,6,14].

Serum IgG4 levels are often within the normal range. Autoantibodies such as p-ANCA or c-ANCA may occasionally be detected, especially in patients with concomitant ulcerative colitis, but they lack diagnostic specificity and are not considered reliable serological markers [3,8,11,15].

Histologically, type 2 AIP is characterised by a pattern called idiopathic duct-centric pancreatitis (IDCP), defined by granulocytic epithelial lesions (GEL) infiltrating the pancreatic duct epithelium, with absent or only scant IgG4-positive plasma cells [6,7,8,9,10,11].

Glucocorticoids are also the treatment of choice and usually induce a rapid response. However, in contrast to type 1 AIP, relapse rates are low (0–10%). Consequently, long-term maintenance therapy is generally not required [3].

#### 2.1.3. Type 3 AIP

This condition is a rare, recently described form of chronic drug-induced inflammatory disease of the pancreas. It results from a T-cell-mediated immune response against the pancreatic ducts and acini, triggered by immune checkpoint inhibitors (ICIs) used in the treatment of advanced malignancies [3,11,16,17,18].

Clinically, it is usually asymptomatic, presenting with painless elevation of serum lipase without a significant increase in IgG4 levels. On computed tomography (CT) and magnetic resonance imaging (MRI), the pancreas usually appears normal or shows only mild signs of interstitial pancreatitis, and a rapid loss of pancreatic volume is typically observed during follow-up [11,16,17,18].

This entity is frequently associated with other ICI-related autoimmune conditions and does not exhibit pathognomonic histopathological features. Treatment is based on discontinuation of ICIs, and neither glucocorticoids nor immunomodulatory or biological therapies appear to play a significant role in its management [16,17,18].

### 2.2. Imaging Evaluation of AIP

#### 2.2.1. CT and MRI

Initial imaging assessment and follow-up of AIP relies primarily on CT and MRI. In both cases, a dynamic contrast-enhanced (DCE) acquisition is recommended, including a pancreatic phase (35–45 s), a portal phase (70 s) and a delayed phase (3–5 min). Other specific sequences that should be included in the MRI protocol are T1-weighted imaging (T1WI) with and without fat suppression, T2-weighted imaging (T2WI) with and without fat suppression, diffusion-weighted imaging (DWI) with apparent diffusion coefficient (ADC) map, and 3D magnetic resonance cholangiopancreatography (MRCP) with maximum intensity projection (MIP) reconstruction [19,20,21].

#### 2.2.2. PET/CT

Another useful technique for diagnosis and follow-up is positron emission tomography with ^18^F-fluorodeoxyglucose (^18^F-FDG PET/CT), which is particularly indicated for identifying extrapancreatic manifestations of IgG4-RD and monitoring response to treatment [2,8,21]. A promising new radiotracer, the ^68^Ga-labelled fibroblast activation protein inhibitor (^68^Ga-FAPI), has also demonstrated high sensitivity in the evaluation of IgG4-RD due to its ability to detect both fibrosis and inflammation. In this regard, recent studies have reported significantly higher uptake of ^68^Ga-FAPI PET/CT compared with ^18^F-FDG PET/CT in affected organs such as the pancreas, bile ducts and lacrimal glands [3,22].

#### 2.2.3. Ultrasound

Conventional ultrasound (US) plays a limited role in the evaluation of AIP, although it may be useful in raising initial clinical suspicion. This technique allows identification of diffuse or focal hypoechoic pancreatic enlargement, as well as involvement of other organs such as the biliary tract, gallbladder and lacrimal glands [23].

#### 2.2.4. Endoscopic Procedures

The imaging techniques described above are usually performed prior to more invasive procedures, such as endoscopic retrograde cholangiopancreatography (ERCP) or endoscopic ultrasound (EUS). Although CT and MRI remain the primary non-invasive imaging modalities for the diagnosis and follow-up of AIP, EUS provides superior spatial resolution for the evaluation of small pancreatic lesions and allows real-time tissue acquisition. Therefore, EUS is generally considered as a complementary technique, particularly useful in indeterminate cases requiring histological confirmation. In this regard, current guidelines recommend EUS-guided fine-needle biopsy (EUS-FNB) in patients with suspected AIP, preferably using a 19-gauge needle, over EUS-guided fine-needle aspiration (EUS-FNA), as it allows acquisition of tissue cores suitable for definitive histopathological diagnosis [2,8,24,25,26].

### 2.3. Presentation Patterns of AIP

**Figure 3:**
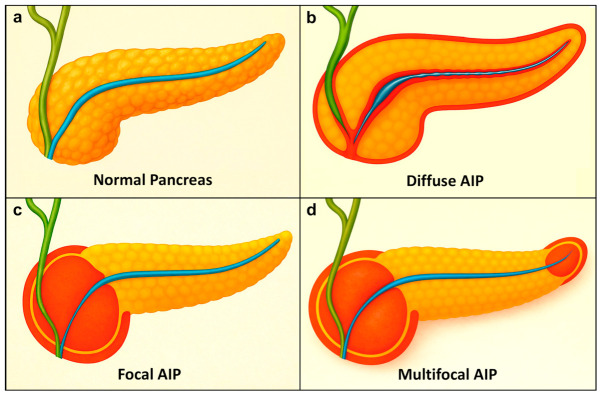
Illustration of the imaging presentation patterns of AIP according to the extent of pancreatic parenchymal involvement. Adapted from Vlachou et al. [20], with permission from RSNA. (**a**) Normal pancreas, with lobulated margins and preserved pancreatic clefts. (**b**) Diffuse AIP pattern, showing an enlarged “sausage-shaped” pancreas with loss of lobulations, a capsule-like “halo” sign, skipped and tapered “icicle-shaped” strictures of the main pancreatic duct (MPD) and stenosis of the distal common bile duct (CBD). (**c**) Focal AIP pattern, presenting as a mass-forming lesion more common in the pancreatic head with focal “halo” sign, causing segmental narrowing of the MPD and intrapancreatic CBD. (**d**) Multifocal AIP pattern, characterised by two or more pancreatic pseudomasses with multisegmental strictures of the MPD and irregular “halo” sign, alternating with areas of spared pancreatic parenchyma.

Although both type 1 and type 2 AIP share similar imaging features, some minor differences have been described. Recent studies suggest that type 2 tends to be more focal and may present with distal shortening of the pancreas due to ductal injury, whereas type 1 is usually diffuse and associated with a greater degree of main pancreatic duct dilatation secondary to compression by inflammatory infiltrate and fibrosis [8,15,19]. From an imaging perspective, three presentation patterns of AIP can be distinguished according to the extent of pancreatic parenchymal involvement [8,20] (Figure 3):

- **Diffuse.** Widespread pancreatic involvement, including the main pancreatic duct (MPD) and the intrapancreatic common bile duct (CBD), represents the most frequent pattern of AIP, accounting for approximately 60–70% of cases.
- **Focal.** Up to one-third of cases present as a focal mass-forming lesion, most often located in the pancreatic head, causing segmental stricture of the MPD and the intrapancreatic CBD without marked upstream dilatation. This pattern of AIP requires differentiation from pancreatic ductal adenocarcinoma (PDAC).
- **Multifocal.** This is the least common pattern and is characterised by multifocal involvement, with areas of spared pancreatic parenchyma in which pancreatic morphology and MPD calibre remain preserved.

### 2.4. Imaging Features of AIP

The distinctive imaging findings of AIP may be diffuse, focal or multifocal, depending on the aforementioned presentation patterns, and they can be grouped into the following categories [3,7,8,12,19,20,21,25,26,27,28,29,30,31,32]:

• **Pancreatic Morphology (CT and MRI):**
  - “Sausage-shaped” pancreatic enlargement with loss of normal clefts and lobulations, usually diffuse and more prominent in the pancreatic tail.
  - Capsule-like “halo” sign, corresponding to a peripancreatic rim of fibrosis and inflammatory infiltrate typically showing low attenuation on CT, low signal intensity on both T1- and T2-weighted MR images and delayed enhancement. It is highly specific for AIP but observed in only around one-third of cases.
• **Ductal Features (best assessed on 3D MRCP):**
  - Long and skipped narrowing of the MPD, often associated with stenosis of the intrapancreatic CBD.
  - “Duct-penetrating” sign, corresponding to uninterrupted visualisation of the MPD traversing the lesion without complete occlusion.
  - “Icicle” sign, representing smoothly tapered strictures of the MPD or its side branches without marked upstream dilatation.
  - “Enhanced-duct” sign, seen as a linear hyperenhancement along the MPD wall due to inflammatory changes.
• **Contrast Enhancement Pattern (CT and MRI):**
  - Reduced uptake of the affected pancreas in the pancreatic phase, sometimes associated with dotted or speckled areas of enhancement representing spared normal parenchyma.
  - Homogeneous delayed enhancement on late phases (3–5 min), a useful feature for differentiation from PDAC (which typically remains hypovascular).
• **Other MRI-Specific Findings:**
  - Hypointensity on T1WI and mild hyperintensity on T2WI of the involved pancreatic parenchyma, reflecting inflammatory changes.
  - Restricted diffusion with low ADC values, which increase after steroid therapy and can therefore be used for treatment monitoring.
• **PET/CT Findings:**
  - Increased FDG uptake in the affected pancreas corresponding to active inflammatory disease, frequently associated with FDG uptake in other organs reflecting synchronous systemic manifestations of IgG4-RD.
• **Other Organ Involvement Secondary to IgG4-RD:**
  - IgG4-SC is the most common extrapancreatic manifestation in patients with AIP. Other systemic manifestations include tubulointerstitial nephritis, retroperitoneal fibrosis, Mikulicz disease, lung disease and aortitis.
• **Post-Treatment Changes After Steroid Therapy:**
  - Resolution of pancreatic enlargement with secondary parenchymal atrophy.
  - Normalisation of the pancreatic enhancement pattern.
  - Disappearance of the capsule-like “halo” sign.
  - Marked improvement or resolution of MPD strictures.

**Figure 4:**
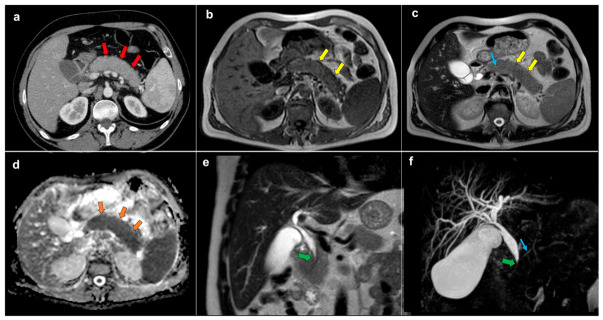
Diffuse AIP in a male patient presenting with painless obstructive jaundice. (**a**) Axial pancreatic-phase CT shows a “sausage-shaped” pancreas with diffuse enlargement and loss of lobulations (red arrows). (**b**) Axial T1- and (**c**) T2-weighted MR images demonstrate a subtle hypointense peripancreatic “halo” (yellow arrows) and a mild diffuse increase in pancreatic T2 signal related to inflammation. Only a short segment of the main pancreatic duct (MPD) is visible on T2WI (blue arrow), due to diffuse stricture caused by fibroinflammatory changes. (**d**) ADC map shows diffusely low values within the pancreatic parenchyma, consistent with restricted diffusion (orange arrows). (**e**) Coronal T2WI and (**f**) 3D MRCP with MIP reconstruction show stenosis and wall thickening of the distal intrapancreatic CBD (green arrow), with moderate upstream biliary dilatation. Note again that only a short segment of the MPD is visible (blue arrow).

**Figure 5:**
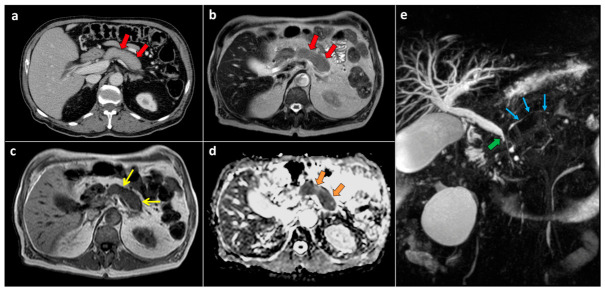
Diffuse AIP in a male patient presenting with painless jaundice and a cholestatic pattern. (**a**) Axial portal-phase CT shows a “sausage-shaped” pancreas with diffuse enlargement and loss of pancreatic clefts (red arrows). (**b**) Axial T2WI demonstrates a mild diffuse increase in pancreatic signal intensity (red arrows). The MPD is not clearly visible due to diffuse stenosis. (**c**) Axial T1WI shows a hypointense peripancreatic capsule-like “halo” (yellow arrows). (**d**) ADC map reveals low values within the pancreatic parenchyma, consistent with restricted diffusion (orange arrows). (**e**) Coronal 3D MRCP with MIP reconstruction demonstrates skipped, smoothly tapered “icicle-shaped” strictures of the MPD (blue arrows), associated with stenosis of the distal CBD (green arrow) and moderate upstream biliary dilatation.

**Figure 6:**
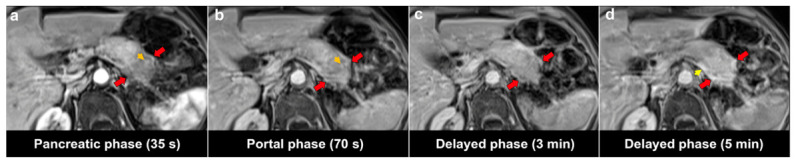
Focal AIP with segmental involvement of the pancreatic body and tail, showing progressive delayed enhancement on dynamic contrast-enhanced MRI. (**a**,**b**) Axial images in the pancreatic and portal phases show reduced uptake in the affected pancreas (red arrows), with dotted or speckled areas of enhancement (orange arrowheads) representing spared normal parenchyma. (**c**,**d**) Axial images in the delayed phases, acquired 3 and 5 min after contrast administration, demonstrate progressive homogeneous enhancement of the involved pancreas (red arrows). Note also the progressive enhancement of the peripancreatic “halo”, which becomes more conspicuous on the 5 min delayed phase (yellow arrowhead).

**Figure 7:**
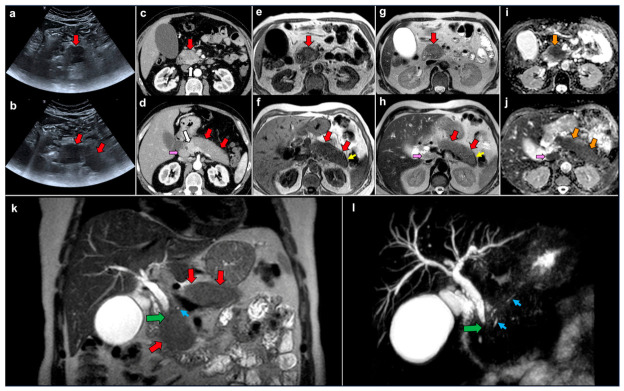
Multifocal AIP in a male patient presenting with epigastric pain and jaundice, showing elevated bilirubin, mildly increased serum amylase and raised serum IgG4 levels. (**a**,**b**) Conventional US shows a hypoechoic pseudomass in the pancreatic head (**a**, red arrow) and hypoechoic enlargement of the pancreatic body and tail (**b**, red arrows). (**c**,**d**) Pancreatic-phase CT demonstrates reduced enhancement of the pancreatic head, body and tail (red arrows), with sparing of normally enhancing parenchyma in the uncinate process and pancreatic neck (white arrows). Note also the presence of an enlarged inflammatory precaval lymph node ((**d**), pink arrow). (**e**,**f**) Axial T1WI shows hypointensity of the involved pancreatic segments (red arrows) and a hypointense capsule-like “halo” ((**f**), yellow arrow). (**g**,**h**) Axial T2WI demonstrates mildly increased signal of the affected segments (red arrows), with a hypointense capsule-like “halo” more evident in the pancreatic tail ((**h**), yellow arrow) and the precaval lymph node (pink arrow). (**i**,**j**) ADC maps show low values in the affected pancreatic segments (orange arrows) and the precaval lymph node (pink arrow). (**k**,**l**) Coronal T2WI and 3D MRCP with MIP reconstruction depict diffuse pancreatic swelling (red arrows), partial visualisation of the MPD (blue arrows) with long skipped strictures, and stenosis of the intrapancreatic CBD (green arrows) with upstream biliary dilatation.

**Figure 8:**
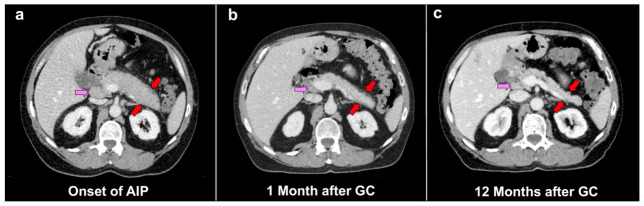
Follow-up CT scans of the previous case of AIP, acquired 1 and 12 months after glucocorticoid therapy, illustrating typical post-treatment changes. (**a**–**c**) Axial portal-phase CT images demonstrate progressive reduction in pancreatic swelling involving the body and tail with secondary parenchymal atrophy, normalisation of the enhancement pattern and gradual disappearance of the “halo” sign (red arrows). The enlarged inflammatory precaval lymph node also shows a decrease in size (pink arrow).

Representative examples of diffuse, focal and multifocal AIP, illustrating the characteristic CT and MRI findings previously described, are shown below (Figure 4, Figure 5, Figure 6, Figure 7 and Figure 8).

### 2.5. Diagnostic Criteria for AIP

**Figure 9:**
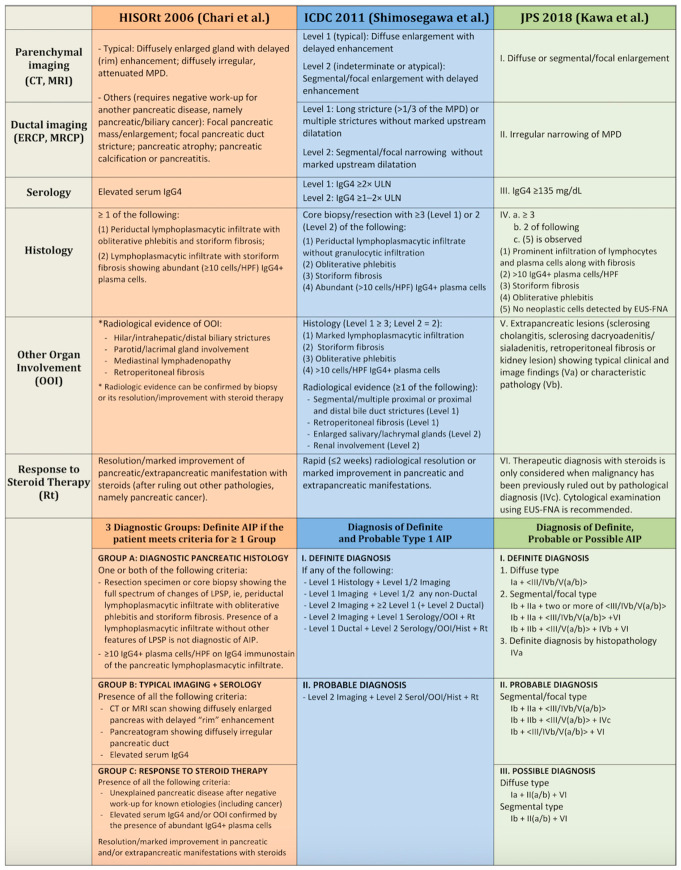
Summary of the main diagnostic criteria available for AIP: HISORt 2006, ICDC 2011 and JPS 2018. Adapted from Chari et al. [33], Shimosegawa et al. [34] and Kawa et al. [35]. MPD = main pancreatic duct; ULN = upper limit of normal value; LPSP = lymphoplasmacytic sclerosing pancreatitis; EUS-FNA = endoscopic ultrasound-guided fine-needle aspiration.

Elevated serum IgG4 levels alone (above the standard threshold of 135 mg/dL) are insufficient to diagnose AIP or IgG4-RD, as this finding is not disease-specific and can be detected in a variety of inflammatory, infectious, allergic and neoplastic disorders. Moreover, serum IgG4 levels may be normal in a substantial proportion of patients with confirmed IgG4-RD. Therefore, an accurate diagnosis of AIP requires a comprehensive approach, integrating clinical presentation, serology, imaging features and histopathological evaluation [1,2,3]. In this regard, several criteria have been proposed over the past two decades by societies in Asia, Europe and North America, aiming to establish reliable diagnostic algorithms for differentiating AIP from PDAC, with particular emphasis on non-invasive methods (Figure 9):

- In 2006, Chari et al. [33] proposed the **Mayo Clinic HISORt criteria** for the diagnosis of type 1 AIP, based on five main diagnostic criteria: histological findings, imaging, serology, involvement of other organs and response to steroid therapy.
- In 2011, Shimosegawa et al. [34] proposed the **International Consensus Diagnostic Criteria (ICDC)** for AIP, a remarkable attempt at standardisation carried out by an international panel of experts. This was the first consensus to enable the diagnosis and comparison of the two main AIP subtypes: type 1 and type 2. According to these criteria, a definitive diagnosis of diffuse type 1 AIP may be established on the basis of clinical, radiological and serological features alone. In cases with atypical mass-forming imaging appearances, and in the absence of other supportive criteria, histological confirmation by surgical biopsy or core biopsy is mandatory to establish a definitive diagnosis and exclude PDAC [3]. These criteria emphasise the need for histological evaluation based on adequate tissue samples, allowing assessment of pancreatic architecture. In current clinical practice, this requirement is most commonly fulfilled by EUS-FNB rather than EUS-FNA.
- In 2020, Kawa et al. [35] published the English version of the **revised diagnostic criteria for AIP of the Japanese Pancreas Society (JPS)**, originally proposed in 2018. Unlike the approach commonly adopted in Western countries, where definitive diagnosis relies primarily on pathological findings obtained by EUS-FNB, the JPS criteria assign greater diagnostic weight to imaging findings from endoscopic retrograde pancreatography (ERP) and magnetic resonance cholangiopancreatography (MRCP). Histological confirmation is reserved for cases in which differentiation from malignancy is challenging, and EUS-FNA is the recommended method for cytological examination. An amendment to these criteria was published in 2022 by Okazaki et al. [32], further defining the key imaging features for differentiating AIP from PDAC on dynamic contrast-enhanced CT and MRI, and acknowledging the increasing diagnostic value of EUS-FNB over EUS-FNA for adequate tissue acquisition and histological assessment.

**Figure 10:**
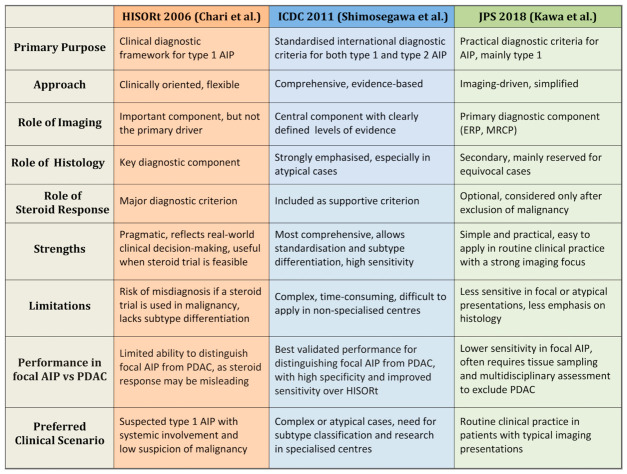
Comparative table outlining the strengths, limitations and preferred clinical settings of the HISORt, ICDC and JPS diagnostic criteria for AIP [33,34,35].

In recent years, several studies have compared the performance of these diagnostic criteria, highlighting relevant differences in their applicability in real-world clinical scenarios [36,37,38,39,40]. However, these systems should not be regarded simply as alternative approaches to diagnosing the same disease, but rather as distinct diagnostic frameworks designed with different primary aims (Figure 10).

The HISORt criteria provide a pragmatic and clinically oriented approach, particularly useful when a steroid trial is being considered, but their reliance on treatment response makes them less suitable when malignancy cannot be confidently excluded. Although historically important as one of the first comprehensive diagnostic frameworks for AIP, they have largely been complemented by the ICDC and, in some settings, the JPS criteria.

The ICDC offer the most comprehensive and standardised framework, with high sensitivity and the added advantage of subtype classification, but their complexity may limit routine use in non-specialised centres. By contrast, the JPS criteria prioritise imaging (ERP and MRCP) and serology, facilitating daily practice, although they may be less sensitive in atypical or focal presentations. In challenging cases, such as focal AIP mimicking PDAC, these differences become particularly relevant, and the criteria are often applied complementarily within multidisciplinary settings to improve diagnostic confidence and guide management.

### 2.6. Differential Diagnosis for AIP

#### 2.6.1. Pancreatic Ductal Adenocarcinoma

PDAC is the most common and lethal form of pancreatic cancer, often presenting with weight loss, progressive jaundice and abdominal pain radiating to the back. Due to its insidious clinical onset, most cases are diagnosed at an advanced stage with vascular invasion and distant metastases. It is most commonly located in the pancreatic head, frequently requiring differentiation from focal AIP. Both conditions may be associated with increased levels of bilirubin and CA 19-9, although marked elevation of this tumour marker is considered a strong indicator of PDAC [41].

**Figure 11:**
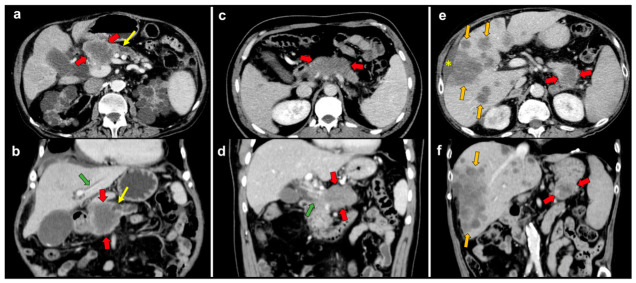
Three representative CT examples of pancreatic ductal adenocarcinoma (PDAC). (**a**,**b**) Axial and coronal pancreatic-phase CT images show a hypovascular mass with hyperenhancing rim in the pancreatic head (red arrows), causing an abrupt cut-off of the MPD with marked upstream dilatation (yellow arrow) and severe atrophy of the pancreatic body and tail. Note also infiltration of the confluence of the superior mesenteric and portal veins and dilatation of the intrahepatic bile ducts (green arrow). (**c**,**d**) Axial and coronal portal-phase CT images show a hypovascular mass involving the pancreatic body and neck, encasing the MPD (red arrows) and causing stenosis of the distal CBD with upstream dilatation (green arrow), as well as thrombosis of the spleno-mesenteric-portal confluence. (**e**,**f**) Axial and coronal portal-phase CT images depict a hypovascular mass with a hyperenhancing rim in the pancreatic tail (red arrows), causing thrombosis of the splenic vein and infiltration of the splenic artery. Note also the presence of multiple liver metastases (orange arrows) and perihepatic ascites (yellow asterisk).

**Figure 12:**
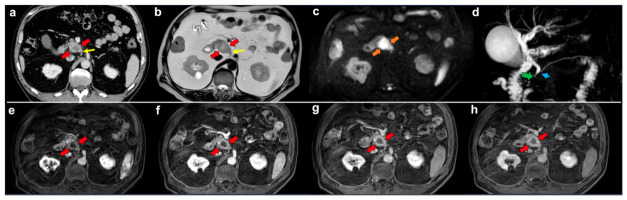
PDAC involving the uncinate process of the pancreatic head, in a male patient with painless jaundice, elevated bilirubin and increased CA 19-9. (**a**) Axial portal-phase CT demonstrates a hypovascular mass with a hyperenhancing rim in the uncinate process (red arrows), with focal infiltration of the adjacent retroperitoneal fat (yellow arrow). (**b**) Axial T2WI shows slightly heterogeneous hyperintensity of the mass (red arrows) and confirms retroperitoneal fat infiltration (yellow arrow). (**c**) DWI (b = 800 s/mm^2^) shows marked restricted diffusion within the pancreatic lesion (orange arrows). (**d**) Coronal 3D MRCP with MIP reconstruction demonstrates stenosis of the distal CBD (green arrow) with moderate upstream biliary dilatation, and focal stricture of the MPD (blue arrowhead) with minimal, regular upstream dilatation. (**e**–**h**) Dynamic contrast-enhanced MRI in the pancreatic (**e**), portal (**f**) and delayed phases acquired 3 and 5 min after contrast administration (**g**,**h**) confirms the presence of a persistently hypovascular mass with a hyperenhancing rim in the uncinate process (red arrows). In this case, the absence of homogeneous delayed enhancement and the presence of infiltrative margins were key findings favouring PDAC over focal AIP. The definitive diagnosis of PDAC was confirmed by EUS-FNA.

On CT and MRI, it usually appears as a poorly defined hypovascular mass with a hyperenhancing rim in the pancreatic and portal phases, showing persistent hypovascularity in delayed phases (in contrast to the late homogeneous enhancement typically observed in AIP). The key ductal feature of PDAC is an abrupt cut-off of the MPD with marked upstream dilatation indicating complete occlusion, in contrast to the “duct-penetrating” sign observed in AIP. It is frequently associated with synchronous dilatation of the CBD, known as the “double-duct” sign. Distal parenchymal atrophy and irregular vascular encasement or thrombosis further support the diagnosis of PDAC (Figure 11 and Figure 12) [8,12,20,21,25,26,27,28,29,30,31,32,41,42,43,44,45].

Other less specific MRI findings for PDAC include hypointensity on T1WI, heterogeneous signal on T2WI and focal restricted diffusion with low ADC values. These ADC values are often higher than those found in AIP, although this is a less reliable feature for differential diagnosis because variable ADC cut-off values have been reported depending on the MRI device [27,32,42,43,44].

^18^F-FDG PET/CT typically demonstrates a single nodular focus of uptake confined to the pancreas (except in metastatic disease), whereas AIP frequently shows multifocal pancreatic and extrapancreatic uptake related to systemic IgG4-RD [32].

**Figure 13:**
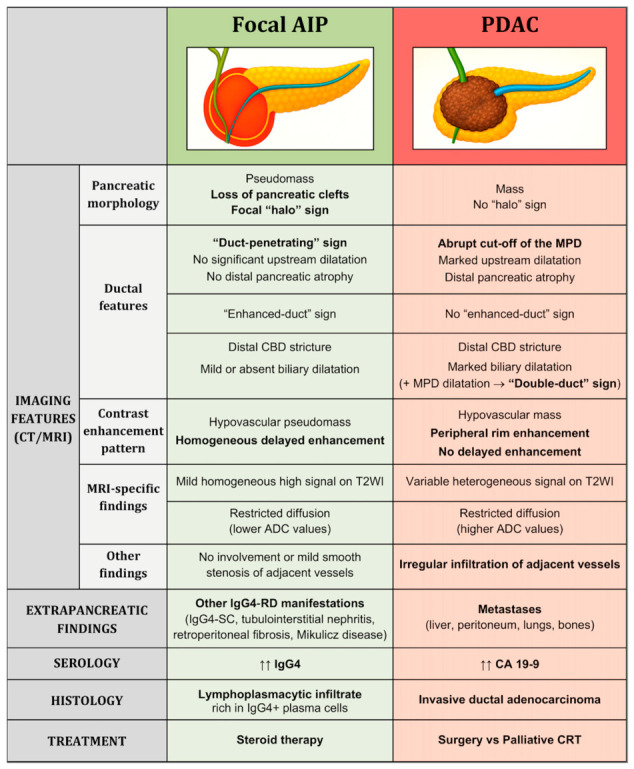
Schematic comparison between focal AIP and PDAC, summarising the key diagnostic features in a structured format. The most discriminative findings are highlighted in bold to facilitate differential diagnosis in clinical practice. Adapted from Khandelwal et al. [8], Tang et al. [31] and Okazaki et al. [32]. MPD = main pancreatic duct; CBD = common bile duct; CRT = chemoradiotherapy.

**Figure 14:**
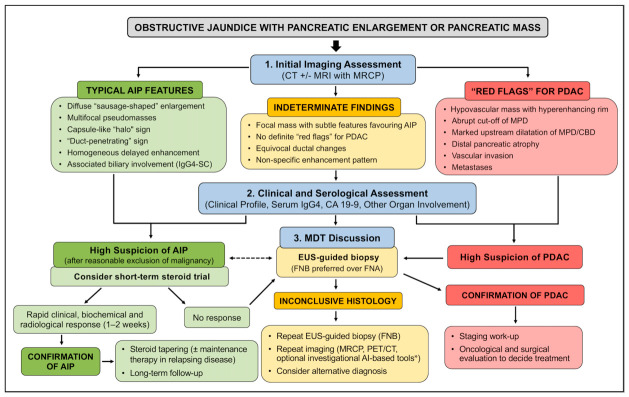
Proposed diagnostic algorithm for the differential diagnosis between AIP and PDAC in patients presenting with obstructive jaundice and pancreatic enlargement or a pancreatic mass. While primarily designed for type 1 AIP, most imaging features and diagnostic approaches remain applicable to type 2 AIP and anicteric presentations. * Note: AI-based tools remain investigational and currently lack sufficient external validation for routine clinical use. AIP = autoimmune pancreatitis; PDAC = pancreatic ductal adenocarcinoma; MPD = main pancreatic duct; CBD = common bile duct; MRCP = magnetic resonance cholangiopancreatography; MDT = multidisciplinary team; EUS = endoscopic ultrasound; FNB = fine-needle biopsy; FNA = fine-needle aspiration; PET/CT = positron emission tomography/computed tomography.

In clinical practice, the differential diagnosis between focal AIP and PDAC is challenging, and relies on integrating clinical presentation, serology and histology with a limited set of imaging hallmarks that favour AIP versus “red flags” suggestive of malignancy (Figure 13). In cases with equivocal imaging findings, histological confirmation by EUS-guided sampling should be strongly considered before initiating steroid therapy. Empirical short-term steroid trials should be approached with caution for selected patients, particularly when malignancy cannot be confidently excluded, due to the risk of delayed cancer diagnosis. Multidisciplinary discussion remains essential in clinical decision-making, guiding management and determining the most appropriate diagnostic and therapeutic strategy. A practical diagnostic algorithm integrating these features is proposed (Figure 14).

#### 2.6.2. Other Causes of Acute and Chronic Pancreatitis

AIP is a rare cause of acute and chronic pancreatitis, which must be differentiated from the more common alcohol- and gallstone-related pancreatitis.

##### Alcohol- and Gallstone-Related Acute Pancreatitis

**Figure 15:**
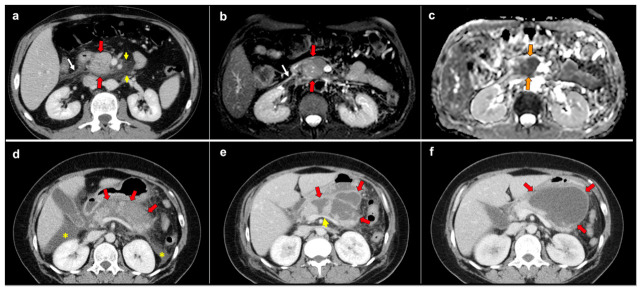
Two representative cases of acute pancreatitis. (**a**–**c**) Focal oedematous pancreatitis in a male patient with acute epigastric pain radiating to the back. (**a**) Axial portal-phase CT shows enlargement of the pancreatic head with slightly heterogeneous enhancement (red arrows), ill-defined stranding of the adjacent fat (yellow arrowheads) and a small amount of fluid in the right anterior pararenal space (white arrow). (**b**) Axial fat-saturated T2WI shows mild hyperintensity of the pancreatic head reflecting inflammatory changes (red arrows) and the right pararenal fluid (white arrow). (**c**) ADC map shows focal restricted diffusion with low ADC values (orange arrows). (**d**–**f**) Necrotising pancreatitis in a female patient with acute abdominal pain. (**d**) Axial portal-phase CT shows diffuse acute pancreatitis with ill-defined areas of hypoenhancing parenchyma due to oedema and glandular necrosis (red arrows), associated with stranding of the adjacent fat and inflammatory exudates extending towards the anterior pararenal spaces (yellow asterisks). (**e**) Follow-up CT obtained 10 days later shows partial replacement of the pancreatic parenchyma and peripancreatic fat by necrotic collections (red arrows), with stenosis of the splenic vein (yellow arrowhead). (**f**) Follow-up CT after 4 weeks shows confluence of the previous collections forming a large pseudocyst abutting the pancreas and stomach (red arrows), which required subsequent drainage by cystogastrostomy.

Acute pancreatitis related to alcohol abuse or gallstones typically presents with severe epigastric pain radiating to the back and vomiting. Jaundice may be present, particularly in biliary forms. Serum lipase and amylase levels are usually elevated, without significant increase in serum IgG4. Imaging typically shows pancreatic enlargement with inflammatory changes and diffuse or focal restricted diffusion, which may overlap with AIP. However, there are additional imaging findings that help in the differential diagnosis (Figure 15) [46]:

- Ill-defined stranding of the peripancreatic fat, in contrast to the well-defined capsule-like rim that may be observed in AIP.
- Peripancreatic inflammatory exudates and fluid collections extending towards the pararenal spaces, which are uncommon in AIP.
- Glandular necrosis and/or peripancreatic fat necrosis, not present in AIP.
- Presence of gas within the pancreas in the emphysematous variant.
- Stenosis of adjacent vessels, most commonly involving the porto-spleno-mesenteric confluence, which may lead to thrombosis.
- Gallstones in the gallbladder and/or the biliary tract, supporting a biliary aetiology.

##### Alcohol-Related Chronic Pancreatitis

**Figure 16:**
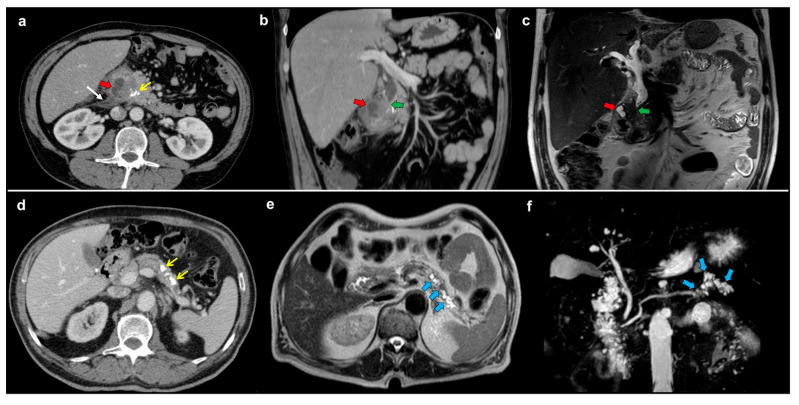
Two examples of chronic pancreatitis on CT and MRI. (**a**,**b**) Axial and coronal pancreatic-phase CT and (**c**) coronal T2WI show findings consistent with chronic groove pancreatitis, including calcifications (yellow arrow), cystic dystrophy of the duodenal wall (red arrow) and marked narrowing of the intrapancreatic CBD (green arrow). Stranding of the adjacent fat with a small amount of free fluid in the right anterior pararenal space is also present (white arrow), suggesting an acute exacerbation. (**d**) Axial portal-phase CT, (**e**) axial T2WI and (**f**) coronal 3D MRCP with MIP reconstruction show diffuse chronic pancreatitis, characterised by parenchymal atrophy, ductal lithiasis and calcifications (yellow arrows), as well as irregular dilatation of the MPD with intermittent strictures (blue arrows).

Chronic mass-forming pancreatitis associated with alcohol abuse exhibits a more insidious clinical onset, which may include dyspepsia, chronic diarrhoea and occasional episodes of epigastric pain radiating to the back, without significant elevation of serum IgG4 levels. In this context, several imaging features may aid in the differential diagnosis with AIP (Figure 16) [21,46]:

- Parenchymal and/or intraductal calcifications (not a typical feature of AIP, although parenchymal calcifications may develop in relapsing disease).
- Presence of pseudocysts (not typical of AIP).
- Irregular dilatation of the MPD with intermittent strictures (rather than the long and smooth “icicle-shaped” narrowing observed in AIP).
- Pancreatic atrophy in advanced stages (which may also be seen in relapsing AIP).
- Chronic thrombosis of adjacent vessels (most commonly involving the splenic vein).

In addition, a focal variant of chronic pancreatitis located in the pancreaticoduodenal groove (“groove pancreatitis”) may be observed, potentially leading to chronic stenosis of the intrapancreatic CBD. Chronic mass-forming pancreatitis can also mimic hypovascular pancreatic neuroendocrine tumours, and contrast-enhanced CT has been evaluated as a useful tool for this differential diagnosis, particularly by analysing tumour attenuation and enhancement patterns across the pancreatic, portal and delayed phases [47].

#### 2.6.3. Pancreatic Lymphoma

Primary pancreatic lymphoma is extremely rare, whereas secondary pancreatic lymphoma is more common, occurring in up to 30% of patients with widespread disease. Clinical symptoms are non-specific and typically include abdominal pain and weight loss. Non-Hodgkin lymphoma, particularly diffuse large B-cell lymphoma, is the most frequent histological subtype, and treatment is based on chemotherapy [48,49].

**Figure 17:**
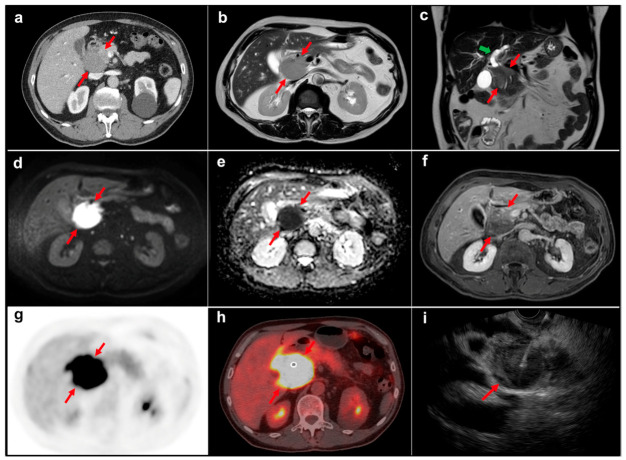
Pancreatic/peripancreatic lymphoma in a male patient presenting with obstructive jaundice. (**a**) Axial pancreatic-phase CT shows a homogeneous soft-tissue mass with mild enhancement in the pancreaticoduodenal groove, infiltrating the pancreatic head (red arrows). (**b**–**f**) Subsequent MRI shows a mildly hyperintense mass on axial and coronal T2WI (**b**,**c**, red arrows), without a capsule-like “halo” sign, infiltrating the intrapancreatic CBD and causing upstream biliary dilatation (**c**, green arrow). The mass shows marked restricted diffusion on DWI (b = 800 s/mm^2^) with corresponding low ADC values (**d**,**e**, red arrows), and mild enhancement on portal-phase DCE-MRI, similar to that observed on CT (**f**, red arrows). (**g**,**h**) ^18^F-FDG PET/CT demonstrates marked hypermetabolism of the mass, with a maximum standard uptake value (SUVmax) of 28 g/mL (red arrows). This constellation of findings favours a lymphoma-related bulky mass over other differential diagnoses such as focal AIP or PDAC. (**i**) Endoscopic ultrasound with fine-needle biopsy (EUS-FNB) of the mass (red arrow) and bone marrow biopsy were performed, with a final diagnosis of Burkitt lymphoma.

On imaging, pancreatic lymphoma most commonly presents as a mildly enhancing, low-attenuation, homogeneous bulky mass on CT, with marked restricted diffusion and low ADC values on MRI. It is most frequently located in the pancreatic head, where it may cause jaundice due to CBD stenosis, mimicking focal AIP and PDAC (Figure 17). In cases of secondary involvement, a diffuse pattern is more common and may closely resemble diffuse AIP, showing pancreatic enlargement without dilatation of the MPD. As an additional confounding factor, pancreatic lymphoma may show a transient response to steroid therapy. Consequently, in most cases a definitive diagnosis cannot be established on the basis of imaging alone, and histological confirmation is required [48,49,50,51]. Nevertheless, there are several imaging features that may be helpful in supporting the diagnosis of pancreatic lymphoma [48,49]:

- Presence of extrapancreatic findings frequently associated with lymphoma (such as organomegaly and multicompartmental lymphadenopathy).
- Absence of other IgG4-related systemic manifestations.
- Absence of the capsule-like “halo” sign.
- Absence of speckled enhancement in the pancreatic phase (often seen in AIP).
- Absence of homogeneous delayed enhancement (a characteristic feature of AIP).

## 3. IgG4-Related Sclerosing Cholangitis

IgG4-related sclerosing cholangitis (IgG4-SC) is a biliary disease of unclear aetiology, associated with elevated serum levels of IgG4. It shows a strong association with AIP, which is present in up to 83–92% of cases. Coexistence with other fibrosing IgG4-related diseases, such as IgG4-related tubulointerstitial nephritis, may also be observed, whereas no association with inflammatory bowel disease has been described [52,53,54,55,56,57,58,59,60].

IgG4-SC is more common in males in their sixth decade of life, and its clinical presentation resembles that of AIP but is often more symptomatic, with marked jaundice, mild-to-moderate right upper quadrant pain and weight loss [52,53,59,60].

Serology typically shows a cholestatic pattern, characterised by elevated bilirubin and raised IgG4 levels (significantly increased in approximately 70–84% of cases), together with a mild-to-moderate increase in CA 19-9 due to biliary stasis and inflammation [52,53,54,55,56,57,58,59,60].

Histology shows dense infiltration of the bile ducts by IgG4-positive plasma cells, with extensive fibrosis and significant periluminal inflammation [55,56,57,58,59,60].

Treatment is based on glucocorticoids. Rituximab and immunomodulators may also be used as alternative treatments in relapsing disease and as maintenance therapy [55,56,57,58,59,60].

### 3.1. Imaging Features of IgG4-SC

Imaging evaluation of IgG4-SC relies on CT and mainly on MRI with MRCP, as the latter provides a better representation of the biliary tree. Typical findings include long, band-like segmental strictures of the bile ducts, most frequently involving the CBD and causing prestenotic dilatation, associated with marked circumferential and symmetrical wall thickening [31,55,56,57,58,59,60]. These strictures are caused by dense lymphoplasmacytic infiltration and fibrosis of the bile duct wall, and the stenotic duct lumen is observed on MRI as a linear hyperintense signal on T2WI. Depending on the location of the strictures, different cholangiographic patterns can be recognised and are described in detail in the diagnostic criteria section.

Endoscopic evaluation is based on techniques such as endoscopic ultrasound (EUS) and intraductal ultrasound (IDUS), which provide an optimal depiction of the concentric bile duct wall thickening. Tissue sampling procedures, such as EUS-FNA and ERCP with brush cytology, remain important for ruling out malignant biliary strictures such as cholangiocarcinoma. However, these techniques are of limited value for establishing a definitive diagnosis of IgG4-SC, and carry a risk of leakage and tumour seeding in case of malignancy [58,59,60].

### 3.2. Diagnostic Criteria for IgG4-SC

**Figure 18:**
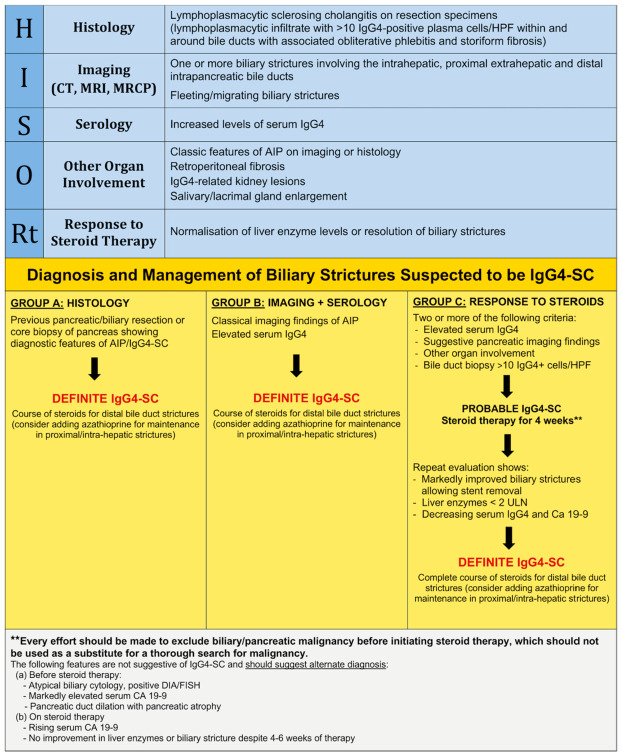
HISORt diagnostic criteria for IgG4-SC (2008). Adapted from Ghazale et al. [55]. HPF = high-power field; ULN = upper limit of normal value; DIA/FISH = digital image analysis/fluorescence in situ hybridization.

Two main sets of diagnostic criteria for IgG4-SC have been proposed to date [55,56,57]. In 2008, a North American group from the Mayo Clinic proposed the HISORt criteria, originally developed for AIP and subsequently adapted for IgG4-SC. These criteria are based on histology, imaging, serology, other organ involvement and response to steroid therapy [55] (Figure 18).

**Figure 19:**
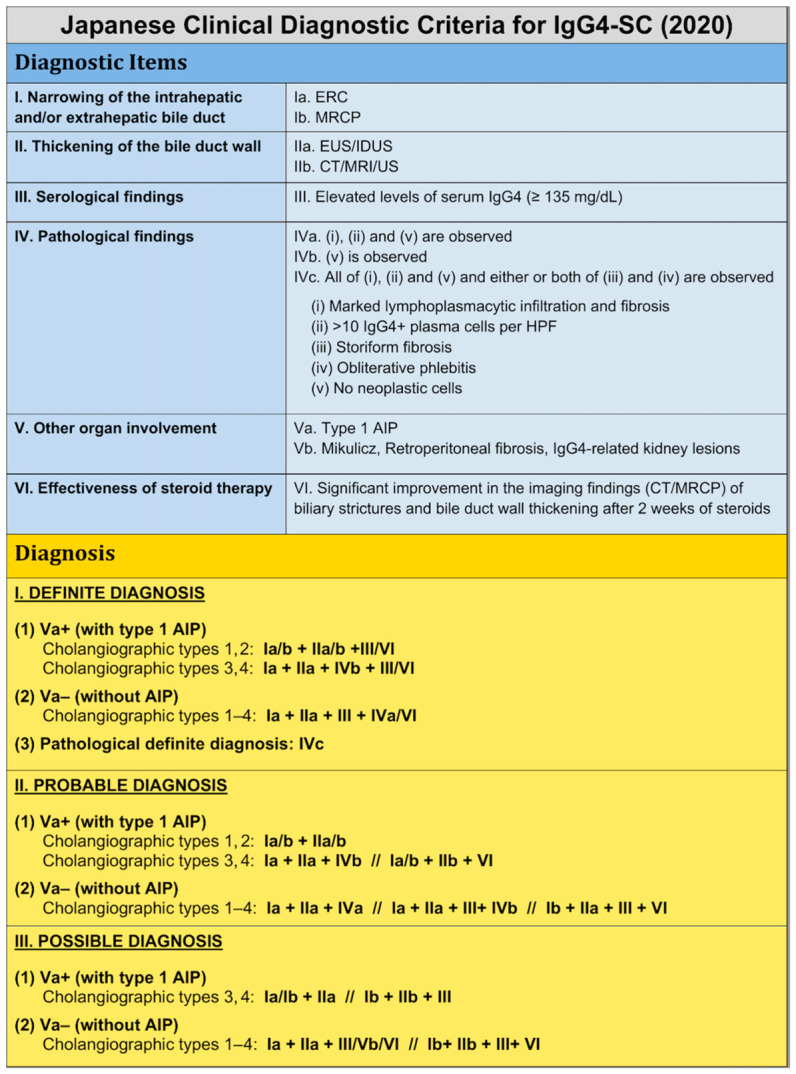
Japanese clinical diagnostic criteria for IgG4-SC (2020). Adapted from Nakazawa et al. [57] and Naitoh et al. [58]. According to these criteria, patients are first classified as “with AIP” or “without AIP”, after which cholangiographic patterns are used to differentiate IgG4-SC from primary sclerosing cholangitis and cholangiocarcinoma. ERC = endoscopic retrograde cholangiography; MRCP = magnetic resonance cholangiopancreatography; EUS = endoscopic ultrasound; IDUS = intraductal ultrasound; AIP = autoimmune pancreatitis; HPF = high-power field.

**Figure 20:**
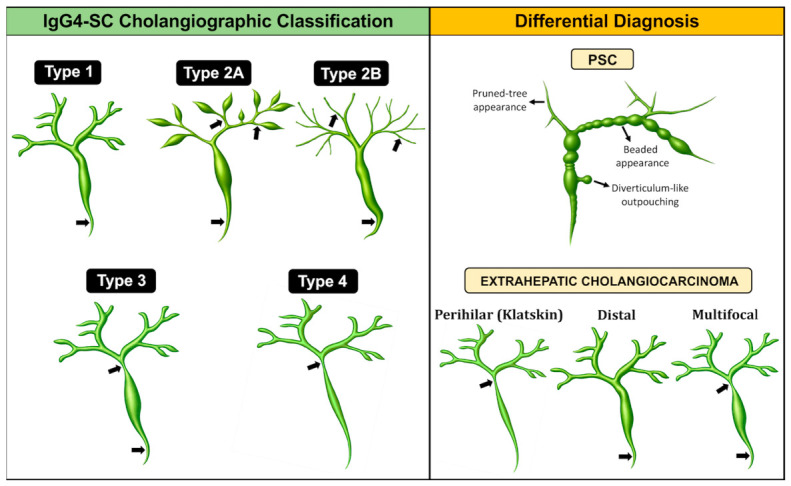
Cholangiographic classification of IgG4-SC based on MRCP findings and its differential diagnosis. Black arrows indicate the location of bile duct strictures. PSC = primary sclerosing cholangitis.

**Figure 21:**
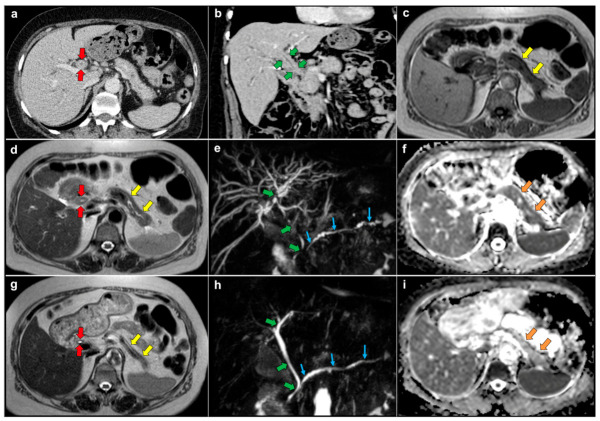
IgG4-related sclerosing cholangitis (IgG4-SC) with concomitant diffuse AIP in a female patient presenting with painless jaundice, elevated bilirubin and increased CA 19-9. (**a**,**b**) Axial and coronal portal-phase CT demonstrates marked concentric and symmetrical thickening of the CBD (red arrows), with a long band-like stricture (green arrows) and upstream dilatation of the intrahepatic bile ducts. (**c**–**f**) Subsequent MRI, including (**c**) axial T1WI, (**d**) axial T2WI, (**e**) coronal MRCP with MIP reconstruction and (**f**) ADC map, confirms the circumferential stenosing thickening of the CBD observed on CT (red arrows). Additionally, segmental strictures of the intrahepatic bile ducts with prestenotic dilatation are observed on MRCP (green arrows), consistent with type 2A IgG4-SC according to the cholangiographic classification. Concomitant signs of diffuse AIP are also present, including a “sausage-shaped” pancreas with a hypointense peripancreatic “halo” best visualised on T1WI (yellow arrows), skipped and tapered “icicle-shaped” MPD strictures (blue arrows) and low ADC values (orange arrows). (**g**–**i**) Follow-up MRI after two months of steroid therapy, including (**g**) axial T2WI, (**h**) coronal MRCP with MIP reconstruction and (**i**) ADC map, demonstrates resolution of both the concentric thickening and narrowing of the CBD (red arrows) and the segmental strictures of the intrahepatic bile ducts (green arrows), without prestenotic dilatation. Marked improvement of the tapered MPD strictures (blue arrows), decreased pancreatic volume with parenchymal atrophy (yellow arrows) and higher ADC values (orange arrows) are also observed.

**Figure 22:**
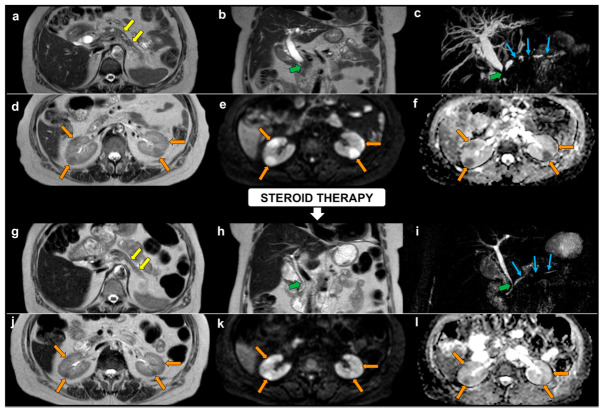
The same patient as in the previous case, a woman with known IgG4-SC and diffuse AIP, who presented one year later with a new episode of painless obstructive jaundice and mild renal function impairment. (**a**–**f**) MRI was performed including (**a**,**b**,**d**) T2WI, (**c**) coronal MRCP with MIP reconstruction, (**e**) DWI (b = 800 s/mm^2^) and (**f**) ADC map. A new severe stricture of the distal CBD with circumferential thickening is seen (green arrow), causing marked upstream biliary dilatation, but on this occasion without stenosis of the intrahepatic bile ducts, consistent with cholangiographic type 1 IgG4-SC. An atrophic pancreas is identified (yellow arrows) due to relapsing AIP, with multisegmental MPD strictures and dilatations (blue arrows). Note also the newly developed bilateral renal cortical lesions, hypointense on T2WI and with marked restricted diffusion (orange arrows), which, in the clinical context, were highly suggestive of IgG4-related tubulointerstitial nephritis. (**g**–**l**) Follow-up MRI after one month of glucocorticoid therapy shows a dramatic response, with resolution of both the concentric thickening and narrowing of the distal CBD (green arrow) and the upstream biliary dilatation, as well as significant improvement of the multisegmental MPD strictures (blue arrows). A significant decrease in size of the renal lesions with mild residual restricted diffusion is also observed (orange arrows), confirming the suspicion of IgG4-related tubulointerstitial nephritis. Post-treatment pancreatic atrophy is evident as well, more severe than on the previous MRI (yellow arrows).

**Figure 23:**
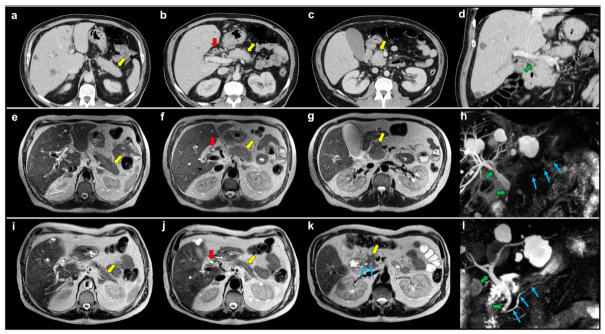
IgG4-SC and diffuse AIP in a male patient presenting with jaundice, elevated bilirubin and right upper quadrant pain. (**a**–**d**) Axial and coronal CT images in the portal phase show signs of diffuse AIP with pancreatic enlargement, loss of lobulations and a subtle hypodense “halo” (yellow arrows). Associated IgG4-SC is evidenced by smooth circumferential wall thickening of the common hepatic duct (CHD) (**b**, red arrow), associated with distal CBD stricture and upstream biliary dilatation (**d**, green arrow). (**e**–**h**) Subsequent MRI was performed. (**e**–**g**) Axial T2WI shows mild hyperintensity of the pancreatic parenchyma with a peripancreatic hypointense “halo” (yellow arrows) and confirms the bile duct wall thickening (**f**, red arrow). Note also the severe strictures of the MPD and intrapancreatic CBD, resulting in poor visualisation of both ducts. (**h**) Coronal 3D MRCP with MIP reconstruction demonstrates hilar stenosis at the biliary confluence and distal CBD stricture (green arrows) with upstream dilatation, consistent with type 3 IgG4-SC. The MPD cannot be clearly identified at the pancreatic head due to severe stenosis, and shows skipped strictures in the pancreatic body and tail, without upstream dilatation (blue arrows). (**i**–**l**) Follow-up MRI after two months of steroid therapy. (**i**–**k**) Axial T2WI shows a decrease in pancreatic volume with resolution of the inflammatory changes (yellow arrows) and improvement of the CHD wall thickening (**j**, red arrow). (**l**) Coronal 3D MRCP with MIP reconstruction demonstrates resolution of both the hilar stenosis at the biliary confluence and the distal CBD stricture (green arrows), accompanied by normalisation of the MPD calibre, which is now clearly visible along its entire course (blue arrows).

In 2012, a Japanese group proposed clinical diagnostic criteria based on characteristic biliary imaging findings, elevated serum IgG4, coexistence with other systemic manifestations of IgG4-RD and typical histopathological features, with response to steroid therapy included as an optional additional diagnostic criterion [56]. These criteria were subsequently revised in 2020 by a group of Japanese researchers specialised in IgG4-SC, who defined the following clinicopathological and imaging items for the definitive diagnosis of IgG4-SC (Figure 19) [57]:

- **Narrowing of the intrahepatic or extrahepatic bile ducts.** On MRCP, IgG4-SC typically presents with diffuse or segmental stenosis of the intrahepatic and/or extrahepatic bile ducts, usually associated with prestenotic dilatation. Stenosis of the intrapancreatic bile duct is observed in almost all cases, most often in association with type 1 AIP [56,57,58]. Based on the cholangiographic appearance and anatomical distribution of these biliary strictures, the Japanese group further subclassified IgG4-SC into four types (Figure 20) [26,56,57,58,59,60,61]:
  (a) Type 1 (64%). Isolated distal CBD stenosis. This is the most frequent presentation and is associated with AIP in the vast majority of cases. Its classification as IgG4-SC remains controversial for some authors, who suggest that this stenosis may be secondary to extrinsic compression of the distal CBD by the inflamed pancreatic parenchyma in patients with AIP. Differential diagnosis includes distal cholangiocarcinoma, PDAC and chronic pancreatitis.
  (b) Type 2 (13%). Distal CBD stenosis associated with intrahepatic bile duct strictures. Two subtypes are recognised, both requiring careful differentiation from primary sclerosing cholangitis (PSC):
    - Type 2A (5%), characterised by segmental intrahepatic strictures with prestenotic dilatation.
    - Type 2B (8%), showing diffuse intrahepatic bile duct strictures with reduced branching, without prestenotic dilatation.
  (c) Type 3 (10%). Combined biliary strictures of the hilar region and distal CBD, which must be differentiated from multifocal extrahepatic cholangiocarcinoma.
  (d) Type 4 (10%). Isolated stenosis of the hilar region, requiring differentiation from perihilar cholangiocarcinoma (Klatskin tumour).
- **Thickening of the bile duct wall.** On CT and MRI, this appears as a long, circumferential and symmetrical wall thickening typically involving both stenotic and non-stenotic segments, a key feature that helps distinguish it from cholangiocarcinoma, in which wall thickening is usually confined to the stenotic areas (Figure 21, Figure 22 and Figure 23) [55,56,57,58,59,60]. On EUS and IDUS, the normal three-layer structure of the bile duct is preserved, with smooth inner and outer margins, which helps distinguish IgG4-SC from PSC and cholangiocarcinoma, in which the layered structure is typically disrupted [59,60]. A bile duct wall thickness > 2.5 mm on MRI has also been proposed as an imaging criterion to distinguish IgG4-SC from primary sclerosing cholangitis (PSC) [2].
- **Serological findings.** Elevated serum IgG4 levels (≥135 mg/dL) are frequently observed but are not specific, as normal values do not exclude IgG4-SC and increased IgG4 concentrations may also occur in other pathological conditions [56,57,58].
- **Pathological findings.** Histopathological examination typically reveals a transmural, diffuse lymphoplasmacytic and eosinophilic infiltrate, associated with storiform fibrosis and obliterative phlebitis.
- **Other organ involvement.** Although IgG4-SC may be associated with a wide spectrum of IgG4-related diseases, these diagnostic criteria are limited to type 1 AIP, Mikulicz disease (dacryoadenitis and/or sialadenitis), retroperitoneal fibrosis and IgG4-related tubulointerstitial nephritis, which may occur synchronously or metachronously with IgG4-SC [54,55,56]. Gallbladder wall thickening due to IgG4-related sclerosing cholecystitis has also been described, but is not included in these criteria [31,56,62].
- **Effectiveness of steroid therapy.** Most patients show a dramatic radiological response to glucocorticoid therapy on CT and MRI. In cases with an insufficient response within 2 weeks, further diagnostic evaluation is mandatory to exclude an underlying malignancy [56,57,58,59,60].

### 3.3. Differential Diagnosis for IgG4-SC

#### 3.3.1. Primary Sclerosing Cholangitis

Primary sclerosing cholangitis (PSC) is a chronic cholestatic disease characterised by progressive inflammation and fibrosis of the bile ducts. It is associated with inflammatory bowel disease in up to 60–80% of cases, most commonly ulcerative colitis. However, this association shows marked geographical variability, with higher prevalence reported in Scandinavian countries and lower rates (around 40%) in Southern Europe and Asia. Several population-based studies have also linked PSC to a significantly increased risk of cholangiocarcinoma and, to a lesser extent, colorectal cancer [53,63,64,65,66,67].

PSC predominantly affects males, accounting for approximately 60–70% of cases, and typically presents in young adults, with a mean age at diagnosis ranging from 35 to 50 years [53,63,64,65,66,67]. Compared with IgG4-SC, the clinical presentation of PSC is usually less acute, and is characterised by a cholestatic biochemical pattern without significant elevation of serum IgG4 levels. Cholestatic pruritus may occur, but jaundice is uncommon in the early stages. Approximately half of patients are asymptomatic at diagnosis and PSC is, therefore, frequently detected incidentally during the evaluation of inflammatory bowel disease [53,63].

**Figure 24:**
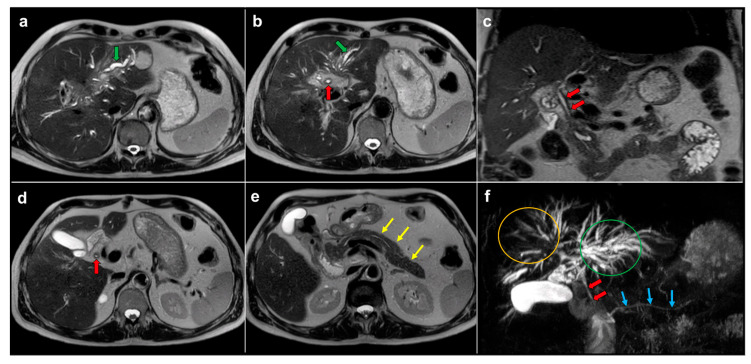
Primary sclerosing cholangitis in a male patient with ulcerative colitis and recurrent episodes of cholangitis. MRI including (**a**–**e**) axial and coronal T2WI and (**f**) coronal 3D MRCP with MIP reconstruction. Circumferential wall thickening and strictures of the CHD and CBD are observed (red arrows), together with short multifocal strictures of the intrahepatic bile ducts, resulting in a “pruned-tree” appearance on MRCP (orange circle). Note also the alternation of normal segments and saccular dilatations (green arrows), producing a “beaded” appearance on MRCP (green circle). The pancreas shows preserved morphology and signal intensity on T2WI (yellow arrows), and the MPD calibre remains normal on MRCP (blue arrows), with no imaging features suggestive of AIP.

Imaging findings are characterised by short multifocal strictures of the intrahepatic and extrahepatic bile ducts, alternating with normal segments and saccular dilatations, resulting in a typical “pruned-tree” and “beaded” appearance, often accompanied by diverticulum-like outpouchings (Figure 24) [53,67,68,69,70]. Some strictures may be longer (band-like) and involve extensive segments of the bile ducts. These features are best depicted on MRI with MRCP, which represents the imaging modality of choice in patients with suspected or established PSC [63,67,69,70].

Histologically, it is characterised by concentric periductal fibrosis, producing the characteristic “onion-skin” appearance around the bile duct epithelium. Progression of this fibrosis leads to obliteration of the bile duct lumen, in a process known as fibrous obliterative cholangitis [71].

PSC is a progressive disease that ultimately leads to biliary cirrhosis and liver failure. To date, no specific medical therapy has demonstrated significant benefit. Immunosuppressants, bile acids, chelating agents and corticosteroids have been used with limited efficacy, although ursodeoxycholic acid is widely prescribed to improve the biochemical parameters of cholestasis. In advanced stages, liver transplantation remains the only curative treatment. However, a high recurrence rate of PSC in transplanted livers has been reported, affecting up to 38% of patients [63].

#### 3.3.2. Cholangiocarcinoma

Cholangiocarcinoma is the second most common hepatobiliary malignancy after hepatocellular carcinoma. It arises from the epithelial lining of the biliary tract and is strongly associated with chronic inflammatory conditions of the bile ducts. The mean age at diagnosis is 60–70 years, with no clear sex predominance [72,73,74].

The clinical presentation may closely resemble that of IgG4-SC, including symptoms such as jaundice, choluria, pruritus, abdominal pain and weight loss. Biochemically, it is typically associated with elevated bilirubin and a marked increase in the tumour marker CA 19-9, without substantial elevation of serum IgG4 levels [72,73,74].

According to its anatomical location, it is classified into three main types [72,73,74,75]:

- **Intrahepatic (10–20%):** Lesion arising from the intrahepatic bile ducts within the liver parenchyma, most commonly mass-forming (although periductal infiltrating and intraductal growing patterns may also be observed). It requires differential diagnosis with hepatocellular carcinoma.
- **Perihilar (Klatskin tumour) (50–60%):** Infiltrative lesion arising from the hilar and perihilar bile ducts (confluence of the right and left hepatic ducts and common hepatic duct). It requires differential diagnosis with type 4 IgG4-SC.
- **Distal (20–30%)**: Infiltrative lesion arising from the CBD, most commonly located in its distal intrapancreatic portion. It requires differentiation from type 1 IgG4-SC.

Perihilar and distal types are considered extrahepatic and may occur in combination as multifocal cholangiocarcinoma, which must be distinguished from type 3 IgG4-SC.

**Figure 25:**
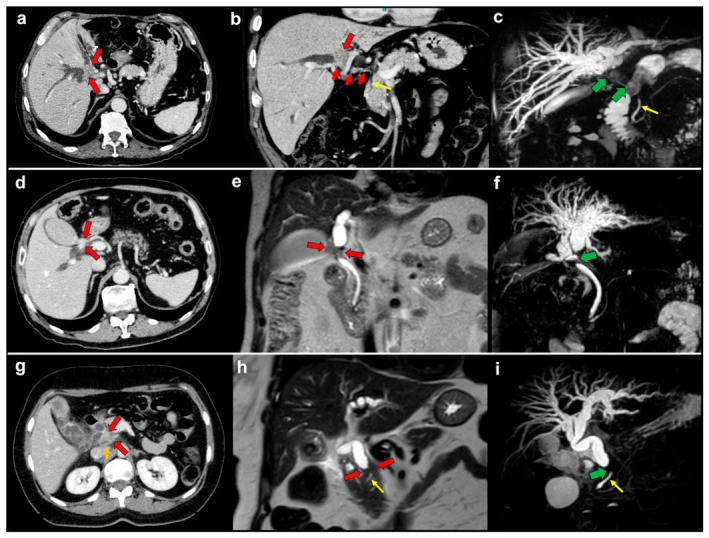
Three illustrative cases of perihilar and distal cholangiocarcinoma on CT and MRI. (**a**–**c**) Perihilar cholangiocarcinoma (Klatskin tumour) in a male patient with epigastric pain, jaundice, and choluria. (**a**,**b**) Axial and coronal portal-phase CT show a slightly hypodense hilar mass encasing the biliary confluence and infiltrating the adjacent hepatic parenchyma (red arrows), with upstream dilatation of the intrahepatic bile ducts. Extensive infiltration of the CHD and upper CBD is best seen on the coronal plane (red arrowheads). (**c**) Coronal 3D MRCP with MIP reconstruction demonstrates abrupt cut-off at the biliary confluence extending towards the CHD and upper CBD (green arrow), with marked upstream biliary dilatation. Note the preserved calibre of the distal CBD (yellow arrow). (**d**–**f**) Perihilar cholangiocarcinoma (Klatskin tumour) in a male patient with jaundice, choluria and acholia. (**d**) Axial portal-phase CT shows a hyperenhancing soft-tissue lesion in the confluence of the CHD and cystic duct (red arrows). (**e**) Coronal T2WI reveals irregular bile duct wall thickening at this level (red arrows). (**f**) Coronal 3D MRCP with MIP reconstruction demonstrates an abrupt cut-off of the distal CHD (green arrow), with significant upstream biliary dilatation. (**g**–**i**) Distal cholangiocarcinoma in a female patient with progressive jaundice, weight loss and right upper quadrant pain. (**g**) Axial portal-phase CT shows a poorly defined, enhancing soft-tissue lesion involving the proximal intrapancreatic CBD (red arrows). A necrotic precaval lymphadenopathy is also evident (orange arrow). (**h**) Coronal T2WI shows irregular soft-tissue thickening encasing the proximal segment of the intrapancreatic CBD (red arrows). (**i**) Coronal 3D MRCP with MIP reconstruction shows abrupt cut-off at this level (green arrow), with marked regular upstream biliary dilatation. The distal prepapillary segment of the CBD shows preserved calibre on both T2WI and 3D MRCP (yellow arrow).

**Figure 26:**
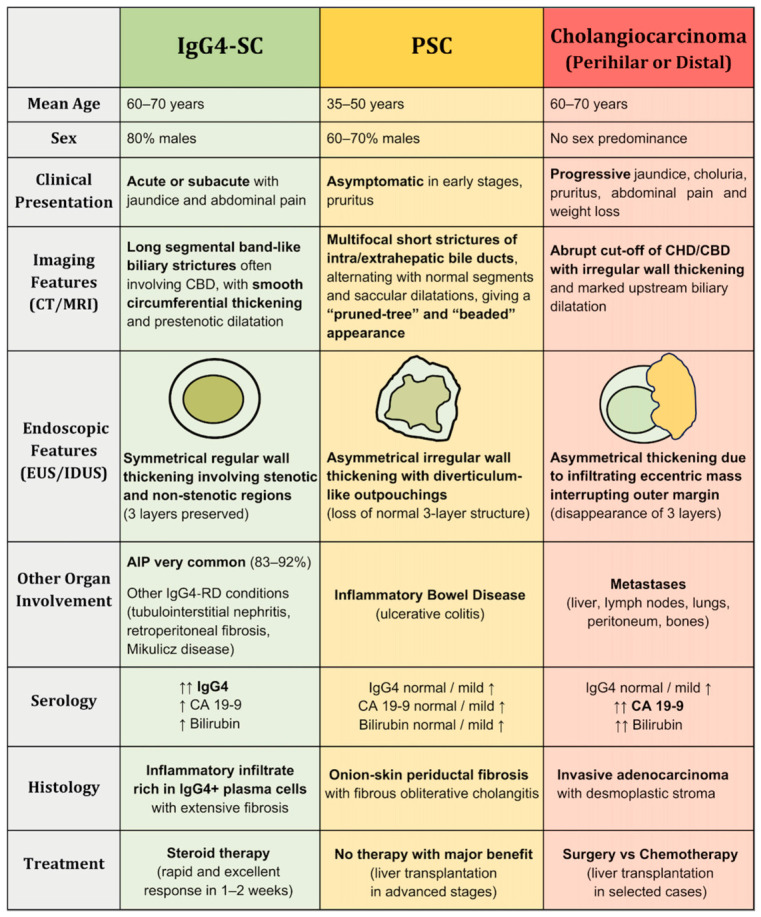
Comparative table summarising the main characteristics of IgG4-SC, primary sclerosing cholangitis (PSC) and extrahepatic cholangiocarcinoma [52,53,54,55,56,57,58,59,60,61,62,63,64,65,66,67,68,69,70,71,72,73,74,75,76]. Key features for differential diagnosis are highlighted in bold. Illustrations adapted from Kersten et al. [59]. This material is distributed under the terms of the *Creative Commons Attribution-NonCommercial 4.0 International License (CC BY-NC 4.0)*. EUS = endoscopic ultrasound; IDUS = intraductal ultrasound; CHD = common hepatic duct; CBD = common bile duct.

Typical CT and MRI features include irregular, asymmetrical thickening of the bile duct wall with delayed enhancement, resulting in focal or segmental strictures and regular upstream dilatation of the intrahepatic bile ducts. Abrupt cut-off of the common hepatic duct (CHD) or common bile duct (CBD) due to complete obstruction is considered a key feature for differentiating cholangiocarcinoma from IgG4-SC and PSC (Figure 25 and Figure 26) [72,73,74]. In this context, careful assessment of associated signs of malignancy, such as enlarged lymph nodes, liver metastases or infiltration of adjacent vessels, should be performed [73].

Histologically, cholangiocarcinoma presents as an adenocarcinoma embedded in a desmoplastic stroma, and is commonly classified into mucinous and mixed subtypes [76]. Surgical resection is the treatment of choice when feasible, and liver transplantation may be considered in selected cases. In unresectable disease, palliative chemotherapy and biliary drainage represent the main therapeutic options. Overall prognosis remains poor, even after surgical resection, with reported 5-year survival rates of approximately 20–40% [76].

## 4. Current Diagnostic Criteria for IgG4-RD

Several diagnostic criteria for IgG4-RD have been proposed by different societies. To clarify these criteria and achieve broader consensus, two major groups (one comprising North American and European experts and the other comprising Japanese experts) have proposed diagnostic algorithms in recent years, in which pancreatic and biliary involvement plays a key role [1,2,3].

### 4.1. ACR/EULAR Classification Criteria for IgG4-RD

**Figure 27:**
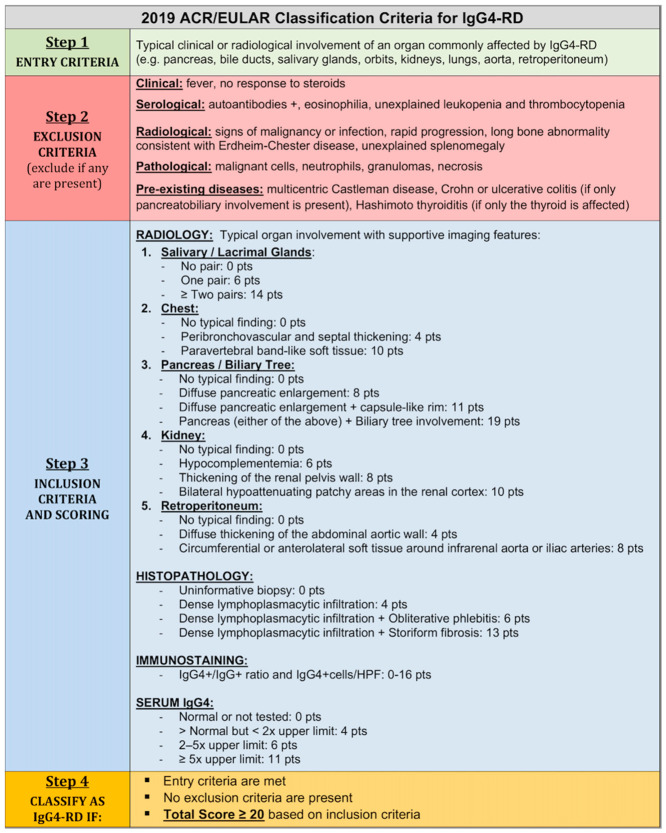
The 2019 ACR/EULAR classification criteria for IgG4-RD. Adapted from Wallace et al. [77]. This classification is based on a four-step process, starting with entry criteria, followed by exclusion criteria and a scoring system derived from the inclusion criteria.

In 2019, the American College of Rheumatology (ACR) and the European League Against Rheumatism (EULAR) proposed the ACR/EULAR classification criteria for IgG4-RD (Figure 27) [77]. These criteria comprise four sequential steps:

- **Step 1: Entry criteria.** Patients with suspected IgG4-RD must show typical involvement of at least one of the most commonly affected organs, supported by clinical, serological, radiological or histopathological findings.
- **Step 2: Exclusion criteria.** A predefined set of exclusion criteria must be assessed. If any exclusion criterion is met, an alternative diagnosis should be considered and the patient cannot be classified as having IgG4-RD.
- **Step 3: Inclusion criteria**. If at least one entry criterion is fulfilled and no exclusion criteria are present, then eight inclusion criteria domains should be scored. Five domains reflect radiological involvement of specific regions (lacrimal and/or salivary glands, chest, pancreas and biliary tree, kidney, retroperitoneum), whereas the remaining three domains cover histopathology, immunostaining and serum IgG4 levels.
- **Step 4: Final classification**. Patients are classified as having IgG4-RD if they meet at least one inclusion criterion, do not meet any exclusion criteria and reach a cumulative inclusion-criteria score of 20 points or higher, which yielded a specificity of 98% and a sensitivity of 82% in the ACR/EULAR validation cohorts. In this scoring system, pancreatic and biliary involvement is highly weighted: 19 points are assigned when both are present, and 8–11 points when only the pancreas is affected [77].

**Figure 28:**
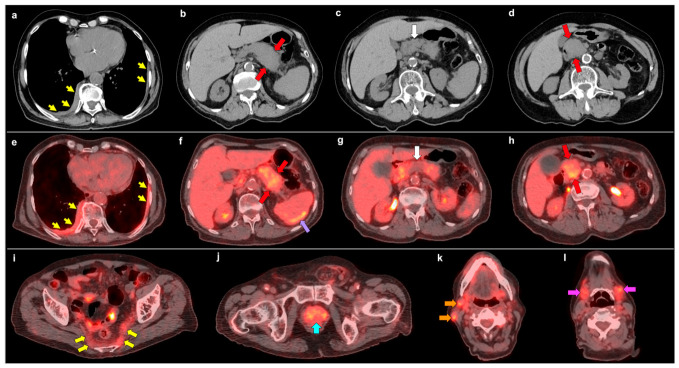
IgG4-RD with multifocal AIP and multiorgan involvement in an anicteric male patient, incidentally detected on non-contrast chest CT performed for persistent cough and recurrent bronchitis. (**a**–**d**) Axial chest CT images show band-like paravertebral soft tissue thickening along the right posterior pleura, and mild thickening of the left pleura (**a**, yellow arrowheads). Inferior sections including the upper abdomen demonstrate enlargement of the pancreatic head, body and tail with loss of lobulations and a pseudomass appearance (**b**,**d**, red arrows), while the pancreatic neck remains preserved (**c**, white arrow). These findings raised suspicion of IgG4-RD with multifocal AIP and pleural involvement, prompting further evaluation with ^18^F-FDG PET/CT and MRI. (**e**–**l**) ^18^F-FDG PET/CT demonstrates intense uptake in the pleural thickening (**e**, yellow arrowheads) and in the pancreatic head, body, and tail (**f**,**h**, red arrows), with sparing of the pancreatic neck (**g**, white arrow). Additional hypermetabolic foci are identified in the perisplenic tissue (**f**, purple arrow), presacral retroperitoneal fat (**i**, yellow arrows), prostate (**j**, cyan arrow), cervical lymph nodes (**k**, orange arrows) and submandibular glands (**l**, pink arrows), consistent with multiorgan involvement secondary to IgG4-RD.

**Figure 29:**
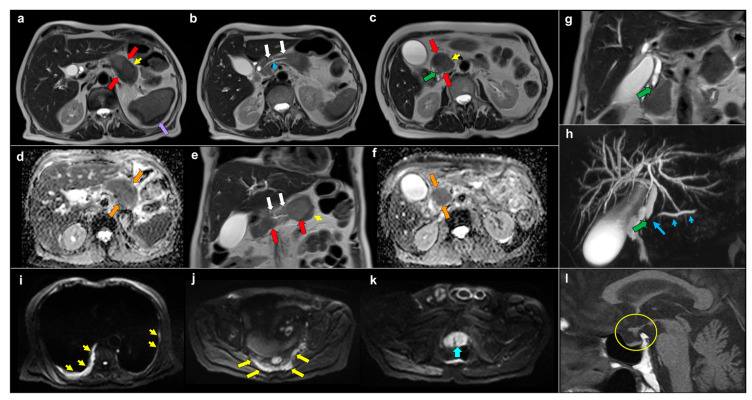
MRI of the previous case, showing multiorgan involvement secondary to IgG4-RD and illustrating the application of the ACR/EULAR classification criteria *. (**a**–**k**) Abdominal MRI including (**a**–**c**,**e**,**g**) axial and coronal T2WI; (**h**) coronal 3D MRCP with MIP reconstruction; (**d**,**f**) ADC maps and (**i**–**k**) DWI (b = 800 s/mm^2^). T2WI show enlargement of the pancreatic head, body and tail with mildly increased signal intensity (red arrows) and corresponding low ADC values (orange arrows). Note also the hypointense “halo” (yellow arrowheads) and the sparing of the pancreatic neck (white arrows). Coronal 3D MRCP demonstrates a tapered stricture of the MPD (blue arrow) with mild upstream dilatation (blue arrowheads), as well as marked stenosis and circumferential thickening of the intrapancreatic CBD (green arrow) with upstream biliary dilatation suggesting type 1 IgG4-SC. Perisplenic soft tissue with low signal on T2WI is also evident (purple arrow). DWI (b = 800 s/mm^2^) shows restricted diffusion in the pleura (yellow arrowheads), presacral fat (yellow arrows) and prostate (cyan arrow), correlating with prior ^18^F-FDG PET/CT findings. (**l**) MRI scan of the pituitary gland was also performed due to suspected hypopituitarism. Sagittal pre-contrast T1WI shows absence of the posterior pituitary bright spot, adenohypophyseal atrophy and marked thinning of the pituitary stalk (yellow circle). In the clinical context, these findings supported the diagnosis of IgG4-related chronic hypophysitis in a fibrotic, involutive stage. * **Application of the ACR/EULAR classification criteria in this case:** This patient showed marked elevation in serum IgG4 levels (5135 mg/dL) and was classified as having IgG4-RD according to the 2019 ACR/EULAR criteria, without the need for histological confirmation. He fulfilled the entry criteria (multiorgan involvement of typical sites: pancreas, salivary glands and chest), met no exclusion criteria and reached a total score of 46 points (submandibular glands = 6 points; pleural soft-tissue thickening = 10 points; diffuse pancreatic enlargement with capsule-like rim plus biliary involvement = 19 points; serum IgG4 > 5× the upper limit of normal = 11 points). Other affected regions (presacral fat, prostate, spleen and pituitary gland) are not included in the organ domains of this scoring system. In contrast, under the 2020 Revised Comprehensive Diagnostic Criteria of the Japanese Pancreas Society, the absence of histopathology would have limited this case to “possible IgG4-related disease” despite typical clinical, radiological and serological findings.

**Figure 30:**
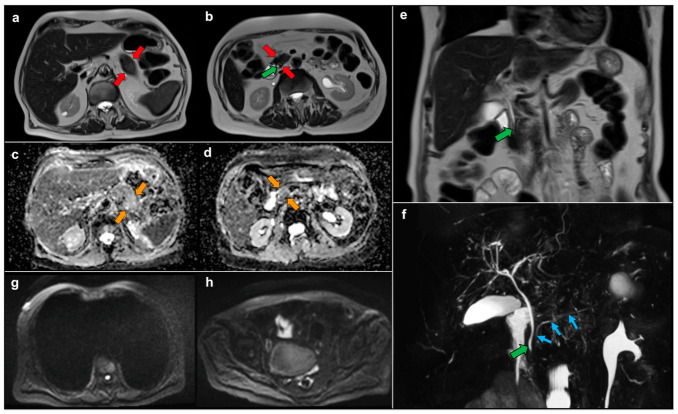
Follow-up MRI of the previous patient (IgG4-RD with multifocal AIP and multiorgan involvement) performed after 2 months of steroid therapy. (**a**,**b**,**e**) Axial and coronal T2WI, (**c**,**d**) ADC maps and (**f**) Coronal 3D MRCP with MIP reconstruction demonstrate marked improvement of the pancreatic fibroinflammatory changes (red arrows), with increased ADC values (orange arrows), as well as resolution of the MPD stricture and mild upstream dilatation (blue arrows). Note also the marked regression of the stenosis and circumferential thickening of the distal CBD (green arrow), without upstream biliary dilatation. (**g**,**h**) DWI (b = 800 s/mm^2^) shows near-complete resolution of the previously observed areas of restricted diffusion in the pleura and presacral fat.

Other groups have evaluated these criteria in real-world settings across different populations, confirming their usefulness and high specificity [78,79,80,81,82,83,84]. Their main advantage over the Japanese criteria is that they allow accurate classification of patients based on characteristic imaging findings, without requiring histopathological confirmation (Figure 28, Figure 29 and Figure 30). A significant limitation is that less frequently affected organs, such as the prostate or the pituitary gland, are not included, and patients without multiorgan involvement may not reach the minimum score. Therefore, a diagnosis of IgG4-RD should not be excluded based solely on these criteria.

### 4.2. Revised Comprehensive Diagnostic (RCD) Criteria for IgG4-RD

**Figure 31:**
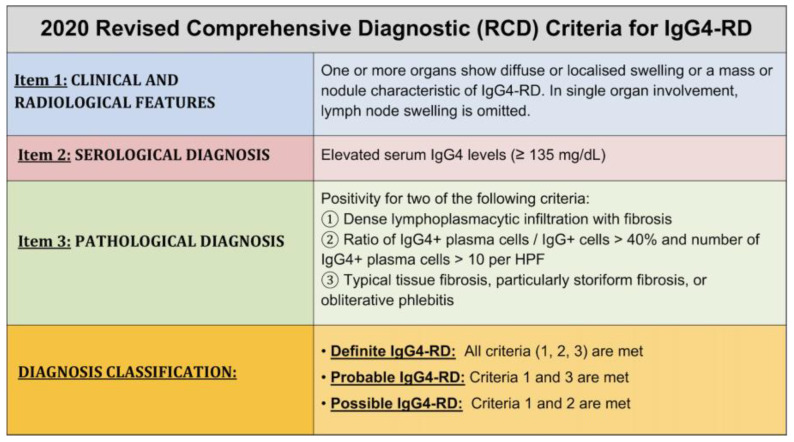
The 2020 Revised Comprehensive Diagnostic (RCD) criteria for IgG4-RD. Adapted from Umehara et al. [85]. These criteria comprise three domains: clinical and radiological features (demonstrating involvement of at least one organ), serological findings (defined by elevated serum IgG4 levels) and histopathological findings.

In 2020, Japanese investigators published the Revised Comprehensive Diagnostic (RCD) criteria for IgG4-RD [85], updating an initial set proposed in 2011 (Figure 31). These criteria comprise three domains: clinical and radiological features (demonstrating involvement of at least one organ), serological findings (elevated IgG4 levels) and histopathological diagnosis. The diagnosis of IgG4-RD is considered “definite” when all three domains are fulfilled, “probable” when the clinical-radiological and pathological criteria are met, and “possible” when only the clinical-radiological and serological criteria are satisfied.

In contrast to the ACR/EULAR classification criteria, which were developed and validated using large international cohorts, the RCD criteria are primarily based on expert consensus. Although they demonstrate very high sensitivity (up to 100%), their specificity is relatively low (approximately 50%), and further validation in independent cohorts is required [86].

## 5. Diagnostic Pitfalls and Interobserver Variability in AIP and IgG4-SC

In daily clinical practice, imaging findings of IgG4-related pancreatobiliary disease frequently overlap with other malignant and inflammatory conditions, leading to diagnostic errors. Mass-forming AIP can closely mimic PDAC and IgG4-SC can simulate PSC or extrahepatic cholangiocarcinoma, particularly in the presence of focal MPD or CBD strictures. Although several imaging features favouring IgG4-RD have been described, such as tapered rather than abrupt MPD stenosis and long band-like biliary strictures, none of these findings are entirely specific.

To further complicate interpretation, some patients with IgG4-related pancreatobiliary disease may have normal serum IgG4 and/or elevated CA 19-9 levels. A transient response to glucocorticoids may also occur in other inflammatory conditions and in neoplastic entities such as pancreatic lymphoma. For this reason, when imaging findings are atypical or malignancy cannot be excluded, tissue sampling and/or close imaging follow-up should be prioritised over empirical steroid therapy [45,60,87,88,89].

Interobserver variability further contributes to these pitfalls, as the assessment of subtle imaging features is subjective and influenced by reader experience and image quality [90]. In this regard, radiology reports should be structured and acknowledge overlapping features, clearly indicating when additional work-up, biopsy or close imaging follow-up is warranted to safely exclude malignant mimickers. This variability also affects other specialists, including endoscopists and pathologists, as some cases remain challenging even after EUS-guided biopsy and histopathological evaluation [91,92].

Systematic application of established diagnostic frameworks may help reduce diagnostic uncertainty in difficult cases, although they cannot fully eliminate it. Consequently, the definitive diagnosis of IgG4-related pancreatobiliary disease should always be based on a multidisciplinary approach integrating clinical, serological, radiological and histopathological data [1,26].

## 6. Emerging Imaging Techniques and Future Directions

Emerging techniques, including quantitative MRI, spectral CT and artificial intelligence (AI)-based tools such as radiomics and multi-omics, are increasingly being explored for diagnostic imaging of pancreatic and biliary disorders. Their main goal is to improve differentiation between benign inflammatory conditions and malignancies, enhancing diagnostic confidence and supporting clinical decision-making within multidisciplinary teams [93,94,95].

### 6.1. Quantitative MRI

A recent meta-analysis by Wang et al. [96] demonstrated the usefulness of quantitative MR imaging biomarkers for distinguishing inflammatory pancreatic masses from PDAC, particularly focusing on the ADC values derived from DWI. These ADC values are typically lower in AIP than in PDAC, but higher in mass-forming pancreatitis.

Another promising quantitative technique for pancreatic tissue characterisation is T1 and, to a lesser extent, T2 mapping. In this regard, PDAC tends to show prolonged native T1 relaxation times compared with chronic pancreatitis and normal pancreatic parenchyma. Active AIP may also demonstrate prolonged T1 relaxation times due to fibroinflammatory changes, although these values may decrease after glucocorticoid therapy. However, current evidence remains limited, and T1/T2 mapping should be regarded as part of the multiparametric MRI approach rather than a standalone tool for differentiating AIP from PDAC [97,98].

### 6.2. Spectral CT

Spectral CT exploits the energy-dependent attenuation of X-rays, enabling material decomposition and providing both morphological and quantitative information beyond conventional CT. Currently, two main technological approaches are available: dual-energy CT (DECT) and photon-counting CT (PCCT). PCCT is a recent evolution that reduces electronic noise and radiation dose, offering detailed anatomical mapping through ultra-high spatial resolution and submillimetre slice thickness [99,100,101].

Both DECT and PCCT provide spectral imaging with virtual non-contrast images, virtual monoenergetic images and iodine maps, improving lesion conspicuity and contrast-to-noise ratio. This results in sharper delineation of poorly defined pancreatic lesions, which may be particularly helpful for the early detection of PDAC [99,100].

Spectral CT also enables quantitative assessment of pancreatic tissue composition, which could potentially assist in differentiating fibroinflammatory changes in AIP from neoplastic tissue in PDAC. However, current evidence remains limited, and further studies are required to establish its clinical utility [99,100,101].

### 6.3. Radiomics and AI

Radiomics enables the extraction of multiple quantitative features, invisible to the naked eye, from images obtained with traditional imaging techniques (CT, MRI, PET/CT, EUS). These features are then translated into high-dimensional data, which are analysed using machine-learning algorithms or more advanced computational methods, such as deep-learning with convolutional neural networks (CNNs) [93,94,95].

In recent years, this novel diagnostic strategy has shown potential to improve (and in some cases surpass) radiologist performance in distinguishing AIP from PDAC. For example, a CT-based radiomic nomogram by Park et al. [102] achieved excellent accuracy of 95.2%, with an area under the receiver operating characteristics curve (AUC) of 0.975. Another retrospective study by Liu et al. [103], using a CT-based deep-learning radiomic model with CNNs, demonstrated strong performance in distinguishing pancreatic cancer tissue from non-cancerous tissue in a multi-ethnic cohort. Notably, this model also showed high accuracy for small pancreatic tumours (<2 cm).

Concerning MRI, a radiomic model based on multiparametric MRI was developed by Deng et al. [104], extracting features from T1WI, T2WI and DCE, to distinguish PDAC from mass-forming chronic pancreatitis, with high diagnostic performance (AUCs of 0.997 in the training internal set and 0.962 in the external validation set).

Regarding PET/CT, studies based on ^18^F-FDG PET/CT images using machine-learning and deep-learning radiomic models [105,106] have also reported a remarkable diagnostic accuracy in differentiating PDAC from AIP (approximately 90%).

Deep learning studies based on EUS images have also shown strong performance in differentiating PDAC from chronic pancreatitis (AUCs ranging from 0.92 to 0.94) [107].

Despite their promising results, the vast majority of these studies remain investigational and have only been tested on single-centre cohorts, lacking standardisation. Therefore, further external validation in large prospective multicentre studies, with diverse real-world populations, is required before they can be routinely implemented in clinical practice.

### 6.4. Multimodality Imaging and Multi-Omics

Despite the promising diagnostic strategies described above, differentiation of focal AIP from small PDAC or mass-forming pancreatitis remains challenging, as imaging and radiomics features often overlap between benign and malignant conditions. For this reason, current and future research is directed towards the development of AI-based fusion models, integrating multimodality imaging data, biochemical markers, clinical information and histopathology into unified diagnostic algorithms, defined under the concept of multi-omics [93,94,95]. For example, Cui et al. [108] developed a multimodal AI model that combines EUS imaging with clinical variables for analysis of solid pancreatic lesions, showing a robust performance in differentiating PDAC from noncancerous lesions (AUCs of 0.996 for the internal dataset and 0.924–0.976 for the external validation datasets) and increasing the diagnostic accuracy of novel endoscopists.

Available imaging techniques for multimodality imaging include, apart from traditional ones like CT, MRI and EUS, hybrid nuclear technologies like PET/CT, PET/MRI and SPECT/CT and emerging optical imaging techniques such as bioluminescence, fluorescence or molecular endoscopic imaging, currently used in preclinical research [109,110]. More recently, other multi-omics data, including proteomics, epigenomics, transcriptomics and metabolomics with novel circulating biomarkers, have also been incorporated into these models [111,112]. The ultimate goal of this holistic approach is to enhance diagnostic accuracy and provide robust evidence to enable personalised treatment strategies, moving towards precision medicine.

## 7. Conclusions

Accurate characterisation of pancreatic and biliary involvement in IgG4-RD is essential, as their frequent synchronous presentation as type 1 AIP and IgG4-SC represents a diagnostic hallmark of this systemic condition. Despite potential overlap with malignancies such as PDAC and cholangiocarcinoma, their distinctive CT and MRI features often allow a confident diagnosis in the appropriate clinical and serological context, within the framework of established diagnostic criteria and multidisciplinary assessment.

AIP typically presents as diffuse pancreatic enlargement with loss of clefts and lobulations, resulting in a “sausage-shaped” morphology. The “halo” sign, although highly specific, is observed in only approximately one-third of cases. In focal forms, homogeneous delayed enhancement and the “duct-penetrating” sign are key imaging features favouring AIP over PDAC, whereas persistent hypovascularity with hyperenhancing rim and abrupt cut-off of the MPD with distal pancreatic atrophy are considered “red flags” for malignancy.

IgG4-SC is strongly associated with type 1 AIP and may be associated with elevated CA 19-9 levels, mimicking PSC and cholangiocarcinoma. Characteristic imaging findings include long, band-like biliary strictures and smooth circumferential wall thickening involving both stenotic and non-stenotic segments.

Both AIP and IgG4-SC show a rapid response to glucocorticoids, and their inclusion in the 2019 ACR/EULAR classification criteria facilitates the diagnosis of IgG4-RD, often obviating the need for histological confirmation.

Looking ahead, AI-assisted imaging is expected to play an increasingly important role in the diagnostic evaluation of IgG4-related pancreatobiliary disease, particularly in improving the differentiation of focal AIP from PDAC through the integration of multimodality imaging, radiomics and multi-omics data into clinically applicable decision-support systems. Although recent studies have shown promising results, further validation in large multicentre cohorts will be required before routine clinical implementation.

In summary, imaging plays a pivotal role in the early recognition of AIP and IgG4-SC, helping to rule out malignancy and supporting timely initiation of steroid therapy, thereby avoiding unnecessary invasive diagnostic procedures and unwarranted surgery.

## Data Availability

No new data were created or analysed in this study. Data sharing is not applicable to this article.

## Abbreviations

The following abbreviations are used in this manuscript:

^18^F-FDG	Fluorine-18 fluorodeoxyglucose
^68^Ga-FAPI	Gallium-68 fibroblast activation protein inhibitor
ACR-EULAR	American College of Rheumatology-European League Against Rheumatism
ADC	Apparent diffusion coefficient
AI	Artificial intelligence
AIP	Autoimmune pancreatitis
AUC	Area under the receiver operating characteristics curve
CBD	Common bile duct
CHD	Common hepatic duct
CNNs	Convolutional neural networks
CT	Computed tomography
DCE	Dynamic contrast-enhanced
DECT	Dual-energy computed tomography
DIA/FISH	Digital image analysis/fluorescence in situ hybridization
DWI	Diffusion-weighted imaging
ERC	Endoscopic retrograde cholangiography
ERCP	Endoscopic retrograde cholangiopancreatography
ERP	Endoscopic retrograde pancreatography
EUS	Endoscopic ultrasound
EUS-FNA	Endoscopic ultrasound-guided fine-needle aspiration
EUS-FNB	Endoscopic ultrasound-guided fine-needle biopsy
HPF	High-power field
IBD	Inflammatory bowel disease
ICDC	International Consensus Diagnostic Criteria
ICI	Immune checkpoint inhibitor
IDUS	Intraductal ultrasound
IgG4	Immunoglobulin G4
IgG4-RD	Immunoglobulin G4-related disease
IgG4-SC	Immunoglobulin G4-related sclerosing cholangitis
JPS	Japanese Pancreas Society
MIP	Maximum intensity projection
MPD	Main pancreatic duct
MRCP	Magnetic resonance cholangiopancreatography
MRI	Magnetic resonance imaging
PCCT	Photon-counting computed tomography
PDAC	Pancreatic ductal adenocarcinoma
PET/CT	Positron emission tomography/Computed tomography
PSC	Primary sclerosing cholangitis
RCD	Revised Comprehensive Diagnostic
SUVmax	Maximum standard uptake value
T1WI	T1-weighted imaging
T2WI	T2-weighted imaging
ULN	Upper limit of normal
US	Ultrasound
